# The Hsp70 co-chaperone Ydj1/HDJ2 regulates ribonucleotide reductase activity

**DOI:** 10.1371/journal.pgen.1007462

**Published:** 2018-11-19

**Authors:** Isaac T. Sluder, Nitika, Laura E. Knighton, Andrew W. Truman

**Affiliations:** Department of Biological Sciences, The University of North Carolina at Charlotte, Charlotte, NC, United States of America; The University of North Carolina at Chapel Hill, UNITED STATES

## Abstract

Hsp70 is a well-conserved molecular chaperone involved in the folding, stabilization, and eventual degradation of many “client” proteins. Hsp70 is regulated by a suite of co-chaperone molecules that assist in Hsp70-client interaction and stimulate the intrinsic ATPase activity of Hsp70. While previous studies have shown the anticancer target ribonucleotide reductase (RNR) is a client of Hsp70, the regulatory co-chaperones involved remain to be determined. To identify co-chaperone(s) involved in RNR activity, 28 yeast co-chaperone knockout mutants were screened for sensitivity to the RNR-perturbing agent Hydroxyurea. Ydj1, an important cytoplasmic Hsp70 co-chaperone was identified to be required for growth on HU. Ydj1 bound the RNR subunit Rnr2 and cells lacking Ydj1 showed a destabilized RNR complex. Suggesting broad conservation from yeast to human, HDJ2 binds R2B and regulates RNR stability in human cells. Perturbation of the Ssa1-Ydj1 interaction through mutation or Hsp70-HDJ2 via the small molecule 116-9e compromised RNR function, suggesting chaperone dependence of this novel role. Mammalian cells lacking HDJ2 were significantly more sensitive to RNR inhibiting drugs such as hydroxyurea, gemcitabine and triapine. Taken together, this work suggests a novel anticancer strategy-inhibition of RNR by targeting Hsp70 co-chaperone function.

## Author summary

Ribonucleotide reductase (RNR) is a key enzyme in the synthesis of DNA and inhibition of RNR leads to cellular sensitivity to radiation. As such, RNR is a well-validated therapeutic target for a variety of diseases including cancer. Anti-RNR drugs are effective but are associated with a range of side effects in patients. Our previous work had identified that the Hsp90 and Hsp70 molecular chaperone proteins regulate RNR. The specificity and activity of Hsp70 and Hsp90 are regulated by “co-chaperone” proteins. We examined RNR activity in cells lacking individual co-chaperones and identified the Ydj1/HDJ2 protein as a novel regulator of RNR in yeast and human cells. Importantly, we demonstrate that inhibiting HDJ2 sensitizes cells to currently used anticancer drugs.

## Introduction

Heat Shock Protein 70 (Hsp70) is a well-conserved, highly expressed molecular chaperone protein. While Hsp70 assists both in the folding of newly synthesized proteins and denatured proteins (“clients”), it also targets damaged proteins for degradation by the proteasomal system [1–3]. Many housekeeping proteins require Hsp70 for stability, making Hsp70 essential for cell viability (2). Cancer cells require Hsp70 to maintain the function of unstable oncoproteins and as such are “addicted” to chaperone function and Hsp70 is often found to be overexpressed in breast and prostate cancers [4–6]. Small molecule inhibitors of chaperones have been developed and assessed for their ability to inhibit cancer cell proliferation *in vivo* and *in vitro*. Despite promising data *in vitro*, chaperone inhibitors have met with limited success in clinical trials due to inherent toxicity of a drug that targets an essential cellular protein [7].

The activity of Hsp70 is regulated by a suite of co-chaperone proteins comprising mainly of Hsp40s and nucleotide exchange factors (NEFs) that assist in the stimulation of ATPase activity and the transfer of clients to Hsp70 for folding [1, 8, 9]. They are a heterogeneous group that can be characterized by the presence of a remarkably conserved 70 amino acid J-Domain [1, 8, 9]. In yeast the major Hsp70 isoform Ssa1 is activated by two related Hsp40s, Ydj1 and Sis1 [10, 11]. Although these two proteins are somewhat functionally redundant, Sis1 is essential for cell viability whereas Ydj1 is not [10, 11]. The study of the behavior of chimeric Ydj1-Sis1 constructs has revealed that the C-terminus of these proteins is the determining factor in client binding and functional distinctiveness [12]. Interestingly, the C-terminus of Ydj1 contains a CAAX farnesylation motif that targets a small population of Ydj1 to the outer surface of the Endoplasmic Reticulum [11, 13]. While both the regulation and function of this targeting remains ill defined, it appears that it is required for interaction with Hsp90 and select client proteins [14]. Ydj1 controls the maturation and stability of a number of Ssa1 client proteins, particularly kinases [15]. Interestingly, Ydj1 also stabilizes several proteins involved in transcription and thus indirectly controls the expression of proteins at the transcriptional level [16, 17].

There are 47 Hsp40s expressed in humans, distributed across the cytoplasm, nucleus, ER and mitochondria and many of these are well conserved with their yeast counterparts [1, 8, 9]. The human homologue of Ydj1 is HDJ2 (also known as DNAJA1), a protein implicated in regulating HIV replication as well as cancer cell growth [18]. Each Hsp40 binds to a specific set of client proteins, thus offering the potential for selective inhibition of tumorigenic vs WT cells [19, 20]. There are no specific Hsp40 inhibitors in clinical trials and it is only recently that Hsp40s have been considered as possible drug targets, possibly due to the lack of characterization of many Hsp40 isoforms. Several dihhydropyrimidines are able to inhibit Hsp40-stimulation of Hsp70 including MAL3-39, MAL3-101 and 116-9e [21]. Recently a novel Hsp40 inhibitor, C86 was identified that promotes androgen receptor degradation offering a novel way to inhibit castration-resistant prostate cancer [22].

Upon DNA damage stress, there is a large remodeling of Hsp70 and Hsp90 complexes and tight association with the ribonucleotide reductase (RNR) complex [23, 24]. The RNR complex catalyzes the production of deoxyribonucleotides (dNTPs) required for DNA repair and S-phase progression. RNR is well conserved between yeast and humans and are comprised of a large (R1) subunit and a small subunit (R2) [25–28]. R1 (R1 in vertebrates, Rnr1/Rnr3 in yeast) forms the catalytic domain while R2 (R2B/R2 in vertebrates, Rnr2/Rnr4 in yeast) acts as regulatory subunits. Although Rnr2 and Rnr4 share sequence homology, only Rnr2 contains the key ligands for tyrosyl radical cofactor [27]. As a result, Rnr2 is essential for cell viability whereas Rnr4 is not. *In vitro* studies have demonstrated a role for Rnr4 in assisting with Rnr2 folding [29]. The fully active RNR complex comprises of a Rnr1 homodimer and Rnr2-Rnr4 heterodimer, with each subunit tightly controlled at the level of expression and cellular localization in response to both cell cycle stage and DNA damage [25]. One of the most highly induced genes in response to DNA damage is a low activity Rnr1 isoform Rnr3. *RNR3* expression is controlled in a Mec1 kinase checkpoint-dependent manner via Crt1 derepression [30, 31]. Because of its high inducibility in response to DNA damage, *RNR3* expression can be used as a measure of DNA damage response pathway activity [32].

RNR is a well-validated anticancer target. Since RNR is required for DNA repair and DNA replication, inhibition of RNR slows cell proliferation and eventually results in S-phase arrest. Hydroxyurea (hydroxycarbamide, HU) was the first small-molecule RNR inhibitor characterized and was approved for clinical use in 1967 [33, 34]. RNR inhibitors are particularly effective when used in conjunction with radiation or other DNA damaging agents [35–37]. Recent studies have demonstrated that pretreatment of cancer cells with either Hsp70 or Hsp90 inhibitors causes destabilization of the RNR complex, sensitizing cells to RNR inhibitors such as HU or gemcitabine [23, 38]. Little is known about the composition of RNR-Chaperone complex, particularly the co-chaperones involved in this process. In this study, we identify Ydj1 and Hdj2 as regulators of RNR activity in yeast and humans, respectively. Moreover, we demonstrate the feasibility of sensitizing cancer cells to RNR inhibitors such as HU by suppressing HDJ2 function.

## Results

### A subset of Hsp70 co-chaperones mediate cellular resistance to hydroxyurea

**Fig 1:**
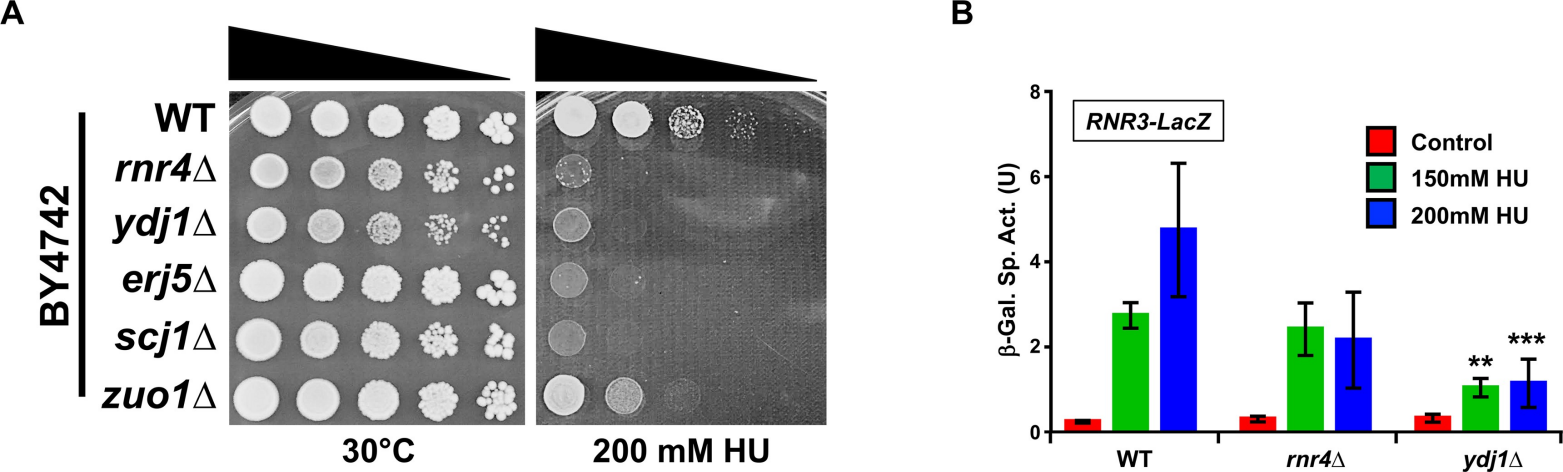
Loss of Ydj1 impairs the DNA damage response. (A) Yeast lacking selected Hsp70 co-chaperones are sensitive to HU. WT BY4742 or BY4742 cells lacking Rnr4, Ydj1, Erjd5, Scj1 and Zuo1 were grown overnight to saturation and serial 10-fold dilutions were plated by pin plating from 96-well plates onto YPD alone or YPD containing 200 mM HU. Plates were imaged after 3 days. (B) Cells lacking Ydj1 are compromised for DNA damage response pathway transcription. An *RNR3-LacZ* reporter plasmid was transformed into the indicated yeast strains. Transformants were grown and subjected to 0, 150mM or 200mM HU for 3 hours. β-Galactosidase activity was measured in crude extracts. β-Galactosidase specific activity (in units) [-Gal Sp. Act. (U)] is shown on the y axis. Each value represents the mean and standard deviation (error bar) from three independent transformants; **, P≤0.01; ***, P≤0.001 as compared to WT cell controls.

Hsp90 and Hsp70 mediate the cellular response to DNA damage by regulating RNR activity. Given that co-chaperones mediate many of the client binding functions in Hsp70 and Hsp90, we sought to uncover candidate co-chaperones that may regulate RNR function. We screened 28 yeast co-chaperone knockout strains (BY4742 background) for cellular sensitivity to Hydroxyurea (HU). While the majority of knockouts showed no significant difference to WT, cells lacking Ydj1, Erj5, Scj1 and to a lesser degree Zuo1 showed increased sensitivity to HU (Fig 1A; S1 Fig). Erj5, Scj1 and Zuo1 are highly specialized co-chaperones that function in the ER and Ribosome respectively. Given that the RNR subunits are not present in either the ER or ribosome under standard conditions, we decided to focus our efforts of understanding the role of Ydj1 on RNR activity.

### Ydj1 regulates the DNA damage response

HU is a potent activator of the DNA damage response pathway, triggering transcription of DNA repair enzymes. In an effort to determine whether Ydj1 controls transcriptional output of the DNA damage response, we compared expression of β-galactosidase driven by a DNA-damage responsive promoter *(RNR3 promoter-lacZ)* in HU-treated WT and *ydj1*Δ cells. Consistent with the increased HU sensitivity of *ydj1*Δ, cells lacking Ydj1 were severely compromised for *RNR3* transcription, suggesting a role for Ydj1 in activation of the DNA damage response (Fig 1B).

### The C-terminus of Ydj1 is required for HU resistance

**Fig 2:**
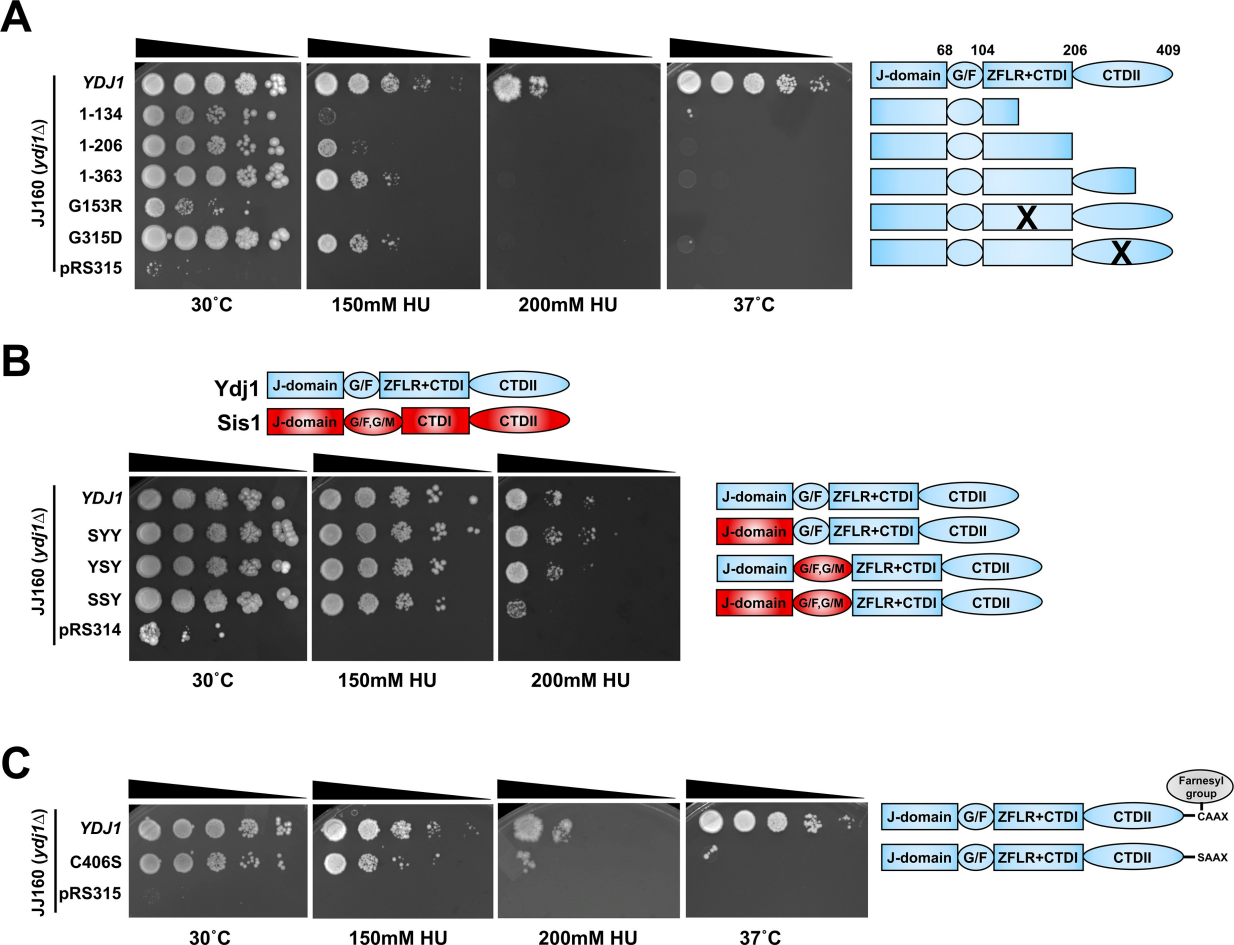
Ydj1 domains required for cellular resistance to HU. (A) The Ydj1 C-terminus is required for HU resistance. JJ160 *ydj1*Δ cells transformed with plasmids encoding full-length Ydj1, truncations of Ydj1 or control plasmid pRS315 were grown to exponential phase, then 10-fold serially diluted onto media containing indicated stressor. Plates were imaged after 3 days. (B) Sis1 and Ydj1 domains are partially interchangeable for HU resistance. JJ160 *ydj1*Δ cells transformed with plasmids encoding full-length Ydj1 or indicated Ydj1-Sis1 fusions were grown to exponential phase, then 10-fold serially diluted onto media containing indicated stressor. Plates were imaged after 3 days. Domains of Ydj1 are colored blue, domains of Sis1 are colored red. (C) Farnesylation impacts Ydj1-mediated HU resistance. JJ160 *ydj1*Δ cells transformed with plasmids encoding WT Ydj1 or farnesylation-deficient mutant C406S were grown to exponential phase, then 10-fold serially diluted onto media containing indicated stressor.

The Ydj1 protein comprises of four functional domains, the N-terminus consisting of the conserved helical J-domain responsible for binding to Hsp70 and stimulating its ATPase activity [8, 39]. In contrast, the C-terminus of Ydj1 consists of two distinct regions, a Zinc-finger like domain and substrate binding domain. These N and C-terminal sections of Ydj1 are tethered together via a flexible linker region rich in glycine and phenylalanine (known as the G/F region). To query the structural requirement for Ydj1-mediated HU resistance, we analyzed an array of Ydj1 C-terminal truncations their ability to suppress the HU growth defect of *ydj1*Δ yeast cells. For these experiments, we utilized JJ160, a *ydj1*Δ strain originating from the stress-sensitive W303 background (S2 Fig. and [16]). The Ydj1 C-terminal truncations displayed a gradual loss of functionality with regard to mediating HU resistance (Fig 2A). While cells expressing Ydj1 lacking the complete or part of the CTDII domain are somewhat sensitive to HU (Ydj1 (1–206) and Ydj1 (1–363)), additional depletion of CTDI as shown for Ydj1(1–134) completely abolished growth at 150mM HU (Fig 2A). Several Ydj1 mutants have been characterized affecting specific facets of Ydj1 function. Two such mutants, G153R and G315D are able to promote GR and ER activity in the absence of hormone when expressed in yeast and are unable to properly fold client proteins such as v-SRC [16, 40]. We examined G153R and G315D for their specific functionality with regard to HU resistance. While cells expressing G315D were moderately sensitive to HU, G153R cells were unable to grow even at 150mM HU (Fig 2A). Taken together, this suggests that both CTDI and CTDII domains play an important role in Ydj1-mediated HU resistance.

### The N-terminus of Sis1 can substitute for Ydj1 in the cellular response to HU

Ydj1 has high homology to another J-protein Sis1. Both possess an N-terminal J domain that binds and regulates Ssa1 [12]. Although highly related, Sis1 and Ydj1 have overlapping yet distinct functions in the cell. Cells lacking Ydj1 are viable, whereas those lacking Sis1 are not [12, 41]. Several studies have attempted to dissect the distinct roles of Ydj1 and Sis1 by creating and expressing Ydj1-Sis1 chimeras [42, 43]. To complement the truncation data in Fig 2B, we asked whether a selection of Ydj1-Sis1 fusions could provide the same function as Ydj1 in mediating the cellular response to HU in yeast. While replacement of either the Ydj1 J-domain or G/F domain with the equivalent Sis1 region alone had little impact, replacement of both resulted in HU sensitivity. This suggests that while the C-terminus of Ydj1 is critical for the response to HU, the N-terminus is also required and that Sis1 and Ydj1 are functionally distinct for this role.

### Farnesylation of Ydj1 is required for the cellular response to HU

While the majority of Ydj1 is localized to the cytoplasm, a fraction exists bound to the cytoplasmic side of the endoplasmic reticulum [13, 44, 45]. This localization is achieved through farnesylation of a “CAAX box” motif on the C-terminus on Ydj1 at C406 [44]. Disruption of Ydj1 farnesylation prevents the interaction of Ydj1 with Hsp90 and client proteins [14]. We queried whether loss of Ydj1 farnesylation impacted cellular resistance to HU. *ydj1*Δ cells expressing either WT Ydj1 or C406S were plated on media containing 150mM or 200mM HU. C406S yeast cells were partially compromised for HU resistance, being more sensitive than WT cells but more resistant than *ydj1*Δ cells to HU (Fig 2C.

### RNR subunit stability is compromised in cells lacking Ydj1

**Fig 3:**
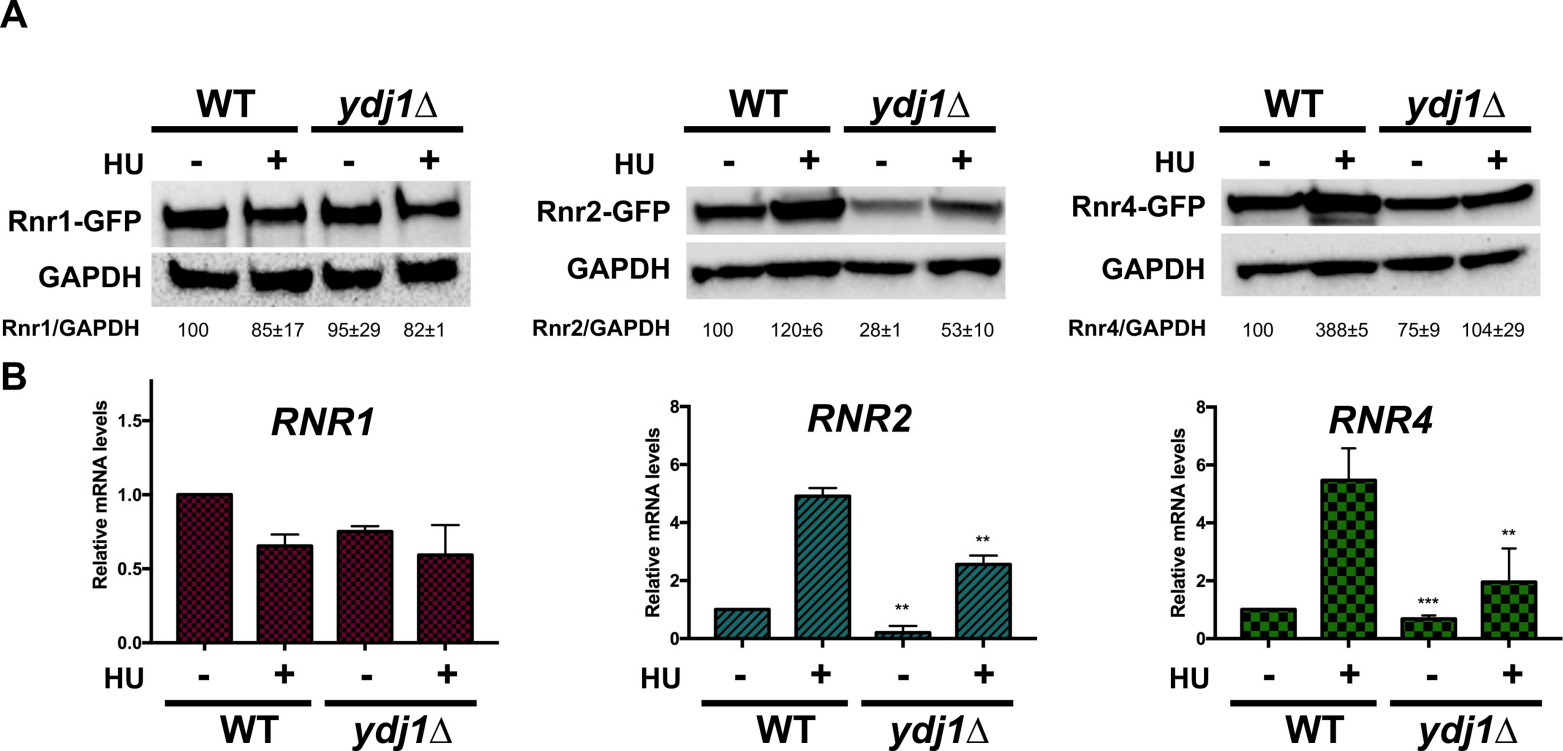
RNR subunit levels in cells lacking Ydj1. (A) BY4742 WT or *ydj1*Δ cells expressing endogenously tagged Rnr1-GFP, Rnr2-GFP or Rnr4-GFP were grown to exponential phase and were either left untreated or were treated with 200mM HU for 3 hours. Cell extracts were obtained, resolved on SDS-PAGE gels and analyzed by immunoblotting with anti-GFP and GAPDH antibodies. (B) Quantitation of *RNR1*, *RNR2* and *RNR4* transcription in WT or *ydj1*Δ cells. Levels of *RNR1*, *RNR2* and *RNR4* mRNAs in BY4742 WT or *ydj1*Δ cells were determined by reverse transcription and RT-qPCR. Signals of *RNR1*, *RNR2* and *RNR4* were normalized against that of *ACT1* in each strain, and the resulting ratios in WT cells were arbitrarily defined as onefold. Data are the average and SD from three replicates.

**Fig 4:**
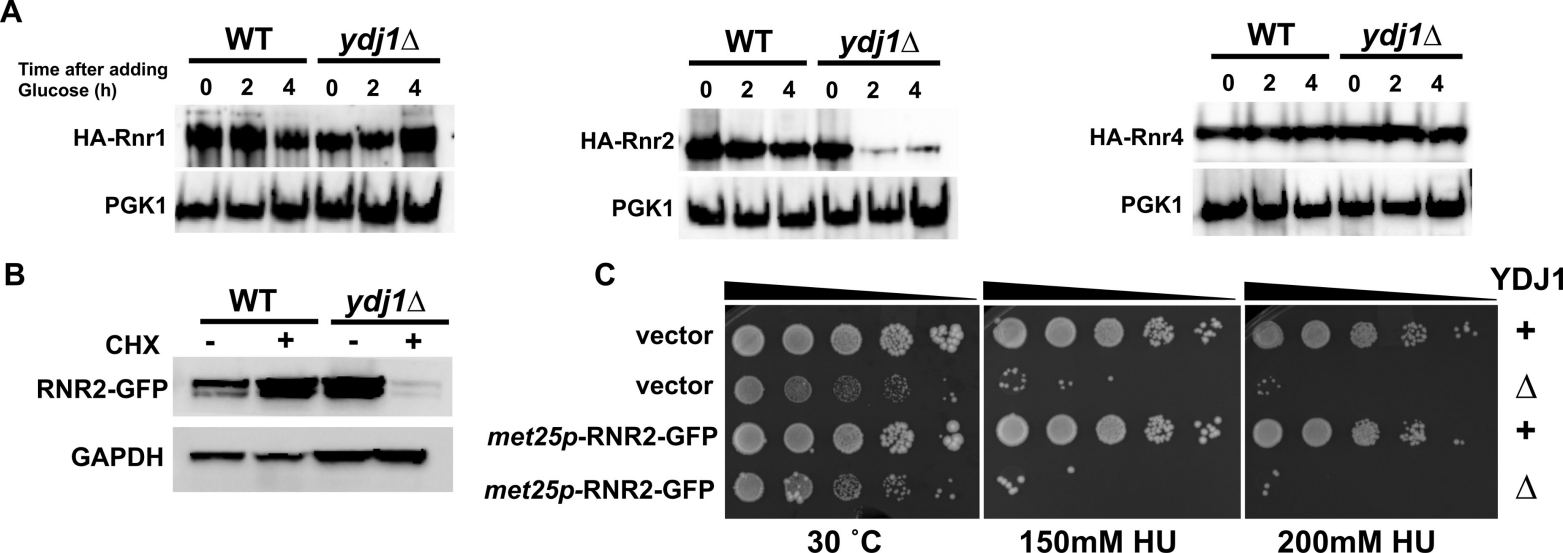
Rnr2 is destabilized in ydj1Δ cells. (A) RNR subunit stability is compromised in cells lacking Ydj1. BY4742 WT or *ydj1*Δ cells transformed with either pGAL1-HA-Rnr1, 2 or 4 plasmids were grown to mid-log phase in YP Galactose medium. Transcription of pGAL1-HA-Rnr1, 2 or 4 was shut off by addition of 2% glucose to cultures. Cell lysates from these samples were analyzed by Western Blotting for stability of HA-RNR subunit (HA antibody) and loading control (PGK1). (B) Examination of Rnr2 stability in WT *ydj1*Δ cells after translational inhibition. BY4742 WT and *ydj1*Δ cells expressing endogenous promoter GFP-tagged Rnr2 were grown to exponential phase in YPD media and then treated with 200 μg/ml cycloheximide for 6 hours to halt protein translation. Cell lysates were obtained and analyzed via Western Blotting for GFP-Rnr2 (GFP antibody) and a GAPDH loading control (GAPDH antibody). (C) Overexpression of Rnr2 does not suppress the HU sensitive phenotype of *ydj1*Δ cells. WT and *ydj1*Δ cells were transformed with either control plasmid pUG36 or *met25p*-RNR2-GFP. Transformants were grown overnight to saturation and serial 10-fold dilutions were plated by pin plating from 96-well plates onto YPD alone or YPD containing 200 mM HU. Plates were imaged after 3 days.

Given that RNR stability is supported by Hsp90 and Hsp70 in yeast and mammalian cells, we wondered whether Ydj1 may be performing a similar role. We integrated GFP-epitope tags onto RNR subunits queried the total levels of Rnr1, Rnr2 and Rnr4 protein in WT and *ydj1*Δ cells. While Rnr1 levels were unchanged, Rnr2 levels were significantly compromised in cells lacking Ydj1 under both unstressed and hydroxyurea treated cells (Fig 3A, S3 Fig). Rnr4 levels were affected in a less dramatic manner; loss of Ydj1 decreased Rnr4 only in HU-treated cells (Fig 3A). Protein levels in cells are balanced by both rate of transcription and protein degradation. Ydj1 has been shown to bind to transcriptional machinery and thus indirectly regulates the transcription of an undetermined number of genes [16, 17]. To determine whether the decreased RNR subunit expression observed in *ydj1*Δ cells was a result of altered transcription, we quantified *RNR1*, *RNR2* and *RNR4* mRNA expression in WT and *ydj1*Δ cells using RT-qPCR. Both *RNR2* and *RNR4* transcription were partially decreased in cells lacking Ydj1 (Fig 3B). This does not appear to be a global effect as the expression of RNR1 remained unchanged in the absence of Ydj1 (Fig 3B). To determine whether the protein stability of Rnr1, Rnr2 and Rn4 had also been compromised *ydj1*Δ cells, we examined the half-life of these proteins by transcriptional shut-off experiments. While Rnr1 and Rnr4 stability were unchanged in *ydj1*Δ cells, Rnr2 stability was substantially lowered (Fig 4A). To corroborate this result, we examined rate of Rnr2 loss in WT and *ydj1*Δ cells treated with cycloheximide, a translational inhibitor. In agreement with the previous result, Rnr2 loss was accelerated in *ydj1*Δ, suggesting increased instability of Rnr2 protein (Fig 4A). We reasoned that if the sole reason for the HU-sensitive phenotype of *ydj1*Δ cells was destabilization of Rnr2, then massive overexpression of Rnr2 may allow *ydj1*Δ cells to grow on HU-containing media. We examined the effect of substantially overexpressing Rnr2 in *ydj1*Δ cells using a multicopy *MET25* promoter driven plasmid (S4A Fig.). Interestingly, cells remained sensitive to HU (Fig 4C). We analyzed the levels of Rnr2 from these cells and still observed a noticeable decrease in Rnr2 levels *ydj1*Δ cells compared to WT (S4B Fig). Taken together, these results imply that the HU sensitivity seen in *ydj1*Δ is a result of decreased Rnr2 and Rnr4 levels, controlled at both the level of transcription and protein degradation.

### Ydj1 interacts with Rnr2 in yeast and humans

**Fig 5:**
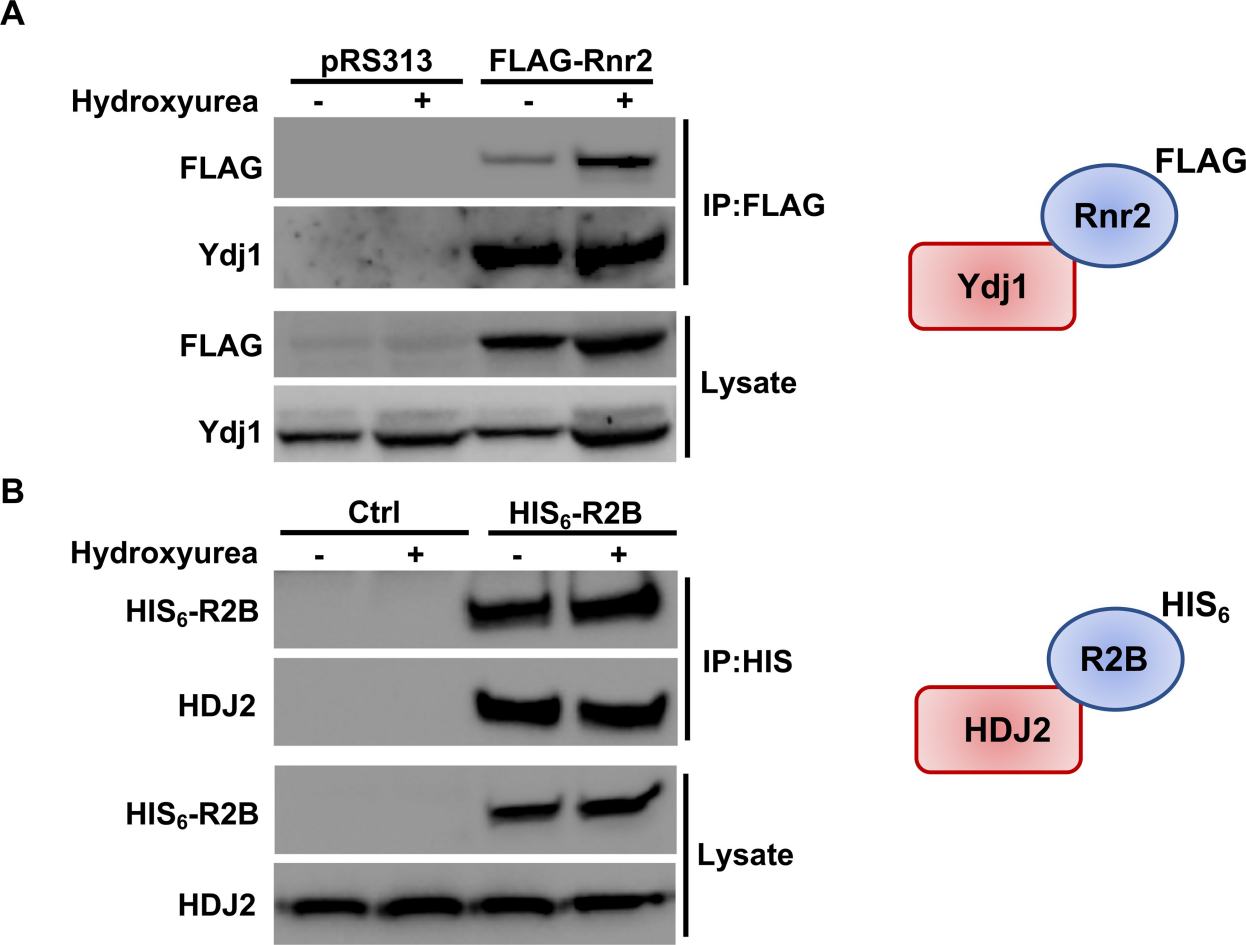
RNR interacts with Hsp40 in yeast and mammalian cells. (A) Rnr2 interacts with Ydj1 in yeast. WT cells transformed with either pRS313 or plasmid expressing FLAG-tagged Rnr2 were grown to exponential phase and were either left untreated or were treated with HU as in Fig 3. Cell extracts (lysate) and immunoprecipitates (IP) with anti-FLAG M2 magnetic beads were subjected to SDS-PAGE and analyzed by immunoblotting with anti-FLAG antibodies to detect Rnr2 or anti-Ydj1 antibodies to detect Ydj1. (B) R2B interacts with HDJ2 in mammalian cells. HEK293 cells were transfected with a plasmid expressing CMV-driven HIS_6_-tagged R2B. Cells extracts were obtained 48 hours post-transfection. Cell extracts (lysate) and immunoprecipitates (IP) with HIS-dynabeads were subjected to SDS-PAGE and analyzed by immunoblotting with tetra-HIS antibodies to detect R2B or anti-HDJ2 antibodies to detect HDJ2.

Previous studies have demonstrated that Hsp90 and Hsp70 bind RNR components [23]. Given that Ydj1 binds both Hsp70 and Hsp90 and plays a role in RNR activity, we sought to determine whether Ydj1 interacted with Rnr2 in yeast. We immunoprecipitated Rnr2 from cells and probed for the presence of Ydj1. Ydj1 was detected as an interactor of Rnr2 in both unstressed and HU-treated cells (Fig 5A). Ribonucleotide reductase is an important chemotherapeutic target in cancer. We considered the possibility that R2B, the human homologue of Rnr2, might similarly interact with HDJ2 (human Ydj1). To examine this, we transfected HEK293 cells with HIS-epitope tagged R2B, purified the R2B complex and probed for associated HDJ2. Interaction of the RNR subunit with HDJ2 was observed, consistent with a conserved role for the co-chaperone (Fig 5B). In contrast to the stress-regulated interaction previously observed between RNR and Ssa1/Hsp82 [23, 24], the interaction between Rnr2/R2B and Ydj1/HDJ2 remained constant between untreated and HU-treated cells (Fig 5A and 5B).

### Hsp70-co-chaperone interaction is important for RNR activity

**Fig 6:**
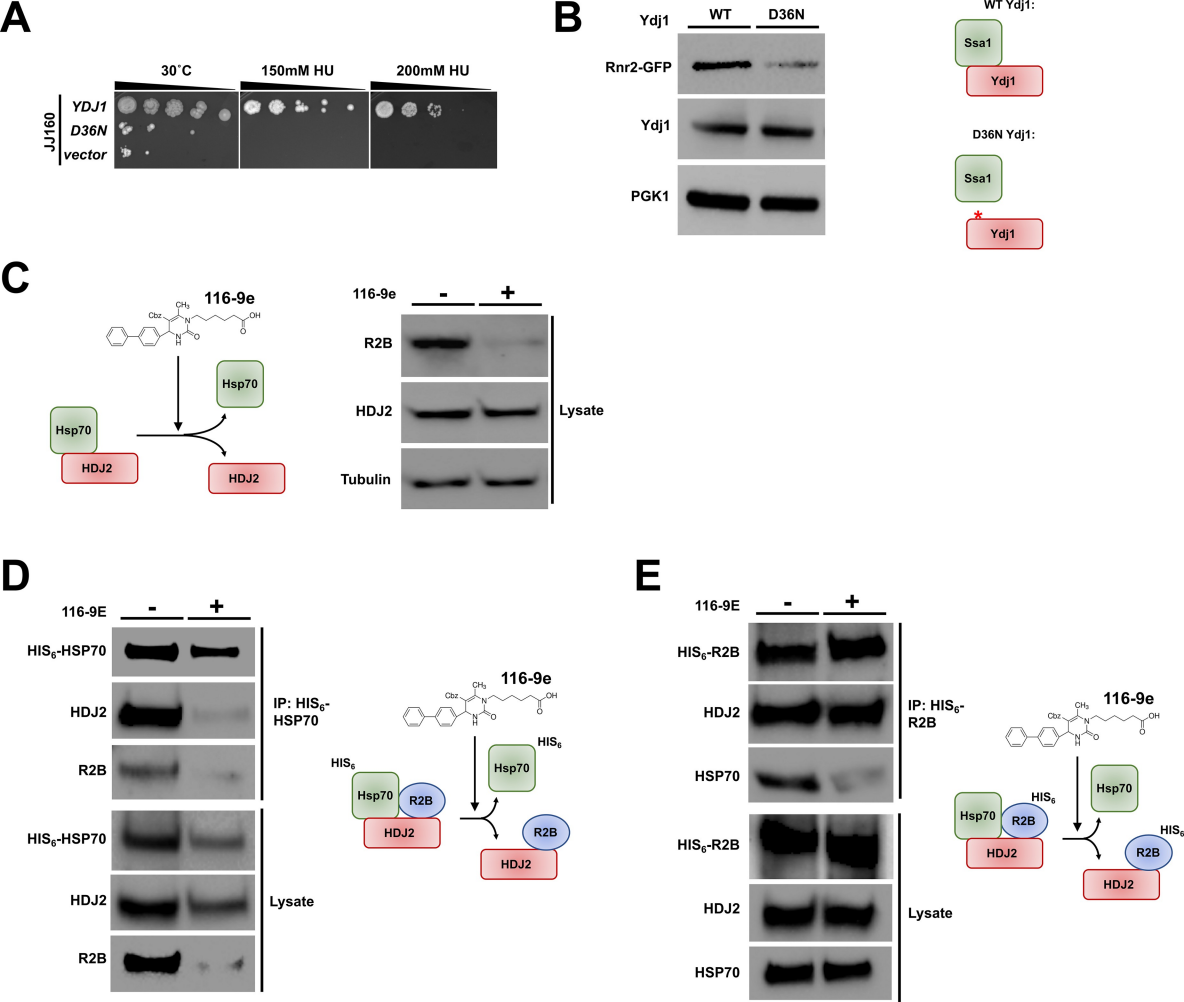
Disruption of the Hsp70-Hsp40 interaction impacts RNR function. (A) Mutation of the HPD motif in Ydj1 sensitizes cells to HU. *ydj1*Δ cells transformed with either a control plasmid, WT YDJ1 plasmid or YDJ1-D36N plasmid were grown overnight to saturation and serial 10-fold dilutions were plated by pin plating from 96-well plates onto YPD alone or YPD containing 200 mM HU. Plates were imaged after 3 days. (B) Mutation of the HPD motif in Ydj1 promotes Rnr2 degradation. *ydj1*Δ *RNR2-GFP* cells transformed with either a WT YDJ1 or YDJ1-D36N plasmid were grown to mid-log phase. Cell extracts were resolved by SDS-PAGE and analyzed by immunoblotting with anti-GFP, anti- Ydj1and anti-GAPDH antibodies. (C) Inhibition of the Hsp70-HDJ2 interaction in cancer cells promotes R2B degradation. HEK293 cells were grown to mid-confluence and then treated with 40 μM 116-9e for 72 hours. Cell extracts were subjected to SDS-PAGE and analyzed by immunoblotting with anti- R2B or anti-GAPDH antibodies. (D) 116-9E disrupts both the HSP70-HDJ2 and HSP70-R2B interaction. HEK293 cells transfected with a plasmid expressing HIS_6_-HSP70 were treated with 116-9E as in (C). HIS-HSP70 complexes were purified from extracts made from these cells, were subjected to SDS-PAGE and were analyzed by immunoblotting with anti-HIS, anti-R2B or anti-HDJ2 antibodies. (E) 116-9E disrupts the R2B-HSP70 interaction but leaves the R2B-HDJ2 interaction intact. HEK293 cells transfected with a plasmid expressing HIS_6_-R2B were treated with 116-9E as in (C). HIS-R2B complexes were purified from extracts made from these cells, subjected to SDS-PAGE and were analyzed by immunoblotting with anti-HIS, anti-R2B or anti-HDJ2 antibodies.

Hsp40-related co-chaperones bind to Hsp70 via conserved J-domains. The J-domain is a 70-amino acid sequence consisting of four helices and a loop region between helices II and III that contains a highly conserved tripeptide of histidine, proline, and aspartic acid (the HPD motif). This region while not required for client protein binding, is absolutely essential for Hsp70-Hsp40 interaction, stimulation of the Hsp70 ATPase and release of substrates post-folding [46, 47]. While several co-chaperones have chaperone-independent activities, Hsp40s typically function through activation of Hsp70. We reasoned that Ydj1’s role in supporting RNR function would occur through its ability to bind Ssa1. We queried whether Ydj1 unable to bind Ssa1 (HPD motif mutant, Ydj1-D36N) could suppress the HU sensitive phenotype of *ydj1*Δ cells. Cells expressing Ydj1-D36N were HU-sensitive, suggesting Ssa1-Ydj1 interaction is critical for RNR activity (Fig 6A). To examine the possibility that the HU sensitivity of Ydj1-D36N cells was caused by lowered Rnr2 abundance, we compared Rnr2 levels in WT Ydj1 and Ydj1-D36N cells. As predicted, Rnr2 levels were lower in cells where the Ssa1-Ydj1 interaction had been disrupted (Fig 6B).

We carried out a parallel experiment in mammalian cells, utilizing a novel small molecule disruptor of Hsp70-Hsp40 interactions, 116-9e [48]. Treatment of HEK293 cells with 116-9e for 72 hours resulted in decreased R2B levels as detected by Western Blot (Fig 6C).

Hsp40s are responsible for delivering client proteins to Hsp70s for folding [8, 49]. To understand how HDJ2-R2B and HSP70-R2B were impacted by 116-9e, we purified either R2B or HSP70 from HEK293 cells treated with 116-9e. While 116-9e promoted the loss of HSP70-R2B and HSP70-HDJ2 interaction, the R2B-HDJ2 interaction persisted (Fig 6D and Fig 6E). These results, taken together suggest the Ssa1-Ydj1/Hsp70-HDJ2 interaction is critical for RNR subunit stability and resistance to RNR inhibiting drugs.

### Inhibition of HDJ2 sensitizes cells to RNR inhibitors

**Fig 7:**
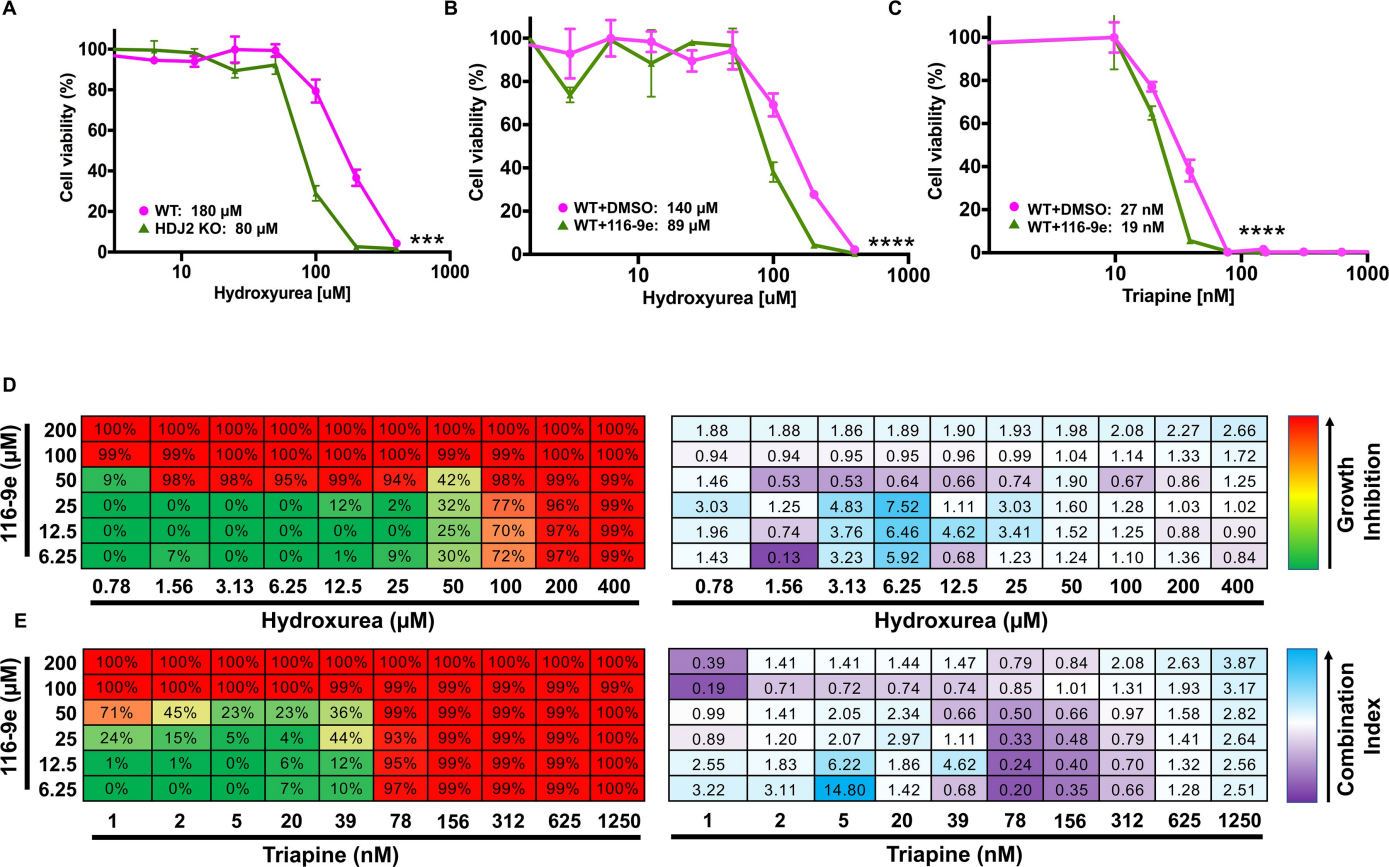
Inhibition of HDJ2 is synergistic with clinically-utilized RNR inhibitors. (A) HDJ2 CRISPR KO cells are more sensitive to HU than WT cells. HAP1 WT and HDJ2 CRISPR KO cells were treated with serial dilutions of HU for 3 days. Cell viability was determined using Celltiter-Glo assay and results shown are average and SD from three replicates (***P<0.001 compared to WT cells, t-test). (B) 116-9e synergizes with HU treatment. HAP1 cells were treated with either DMSO (control) or 116-9e for 24 hours in combination with serial dilutions of HU for 3 days. Data are the average and SD from three replicates (****P<0.0001 compared to HU only treated cells, t-test). (C) 116-9e synergizes with triapine treatment. HAP1 cells were treated with either DMSO (control) or 116-9e for 24 hours, then further treated with serial dilutions of triapine for 3 days. Data are the average and SD from three replicates (***P<0.0001 compared to triapine treated cells, t-test). (D) Combination assay for 116-9e and HU. HAP1 cells were treated with combinations of 116-9e and HU for 72 h and growth inhibition was determined using CellTiter-Glo assay. Combination Index (CI, measure of drug synergy) was determined using Chou-Talalay method via Compusyn software. CI values of <1 indicate drug synergy. (E) Combination assay for 116-9e and triapine. HAP1 cells were treated with combinations of 116-9e and triapine for 72 h and growth inhibition was determined using CellTiter-Glo assay. Combination Index (CI, measure of drug synergy) was determined using Chou-Talalay method via Compusyn software. CI values of <1 indicate drug synergy.

Having established that either loss of Ydj1/HDJ2 compromises RNR activity in both yeast and mammalian cells, we considered whether this regulation might be used to sensitize cancer cells to HU. We examined the difference in drug sensitivity of WT HAP1 cells compared to HAP1 HDJ2 knockout cells created via CRISPR disruption. HDJ2 KO cells were markedly more sensitive than WT cells, with a 60% decrease in IC_50_, from 180μM to 80μM (Fig 7A). These results suggested that 116-9e (an inhibitor of HDJ2) may be synergistic with HU. We queried the difference in drug sensitivity of cells exposed to HU and DMSO compared to cells exposed to a combination of 116-9e and HU. The combination treatment was highly synergistic, promoting a decrease in apparent IC_50 of_ HU from 140μM to 89μM (Fig 7B). While HU has been widely utilized as an anticancer drug it has a short half-life in the body, relatively low affinity for RNR and cells tend to resistance over time. Triapine is a next generation RNR inhibitor, possessing high potency in cell and enzyme-based assays. We examined the difference in drug sensitivity of cells exposed to triapine and DMSO compared to cells exposed to a combination of 116-9e and triapine. The combination treatment was also synergistic, promoting a decrease in apparent IC_50 of_ HU from 27nM to 19nM (Fig 7C). To determine synergy in a more quantitative manner, we calculated drug synergy (CI values) between 116-9e and either HU or triapine across a broad range of concentrations. In both cases, 116-9/HU and 116-9e/triapine demonstrated significant synergy (CI<1) across a range of doses (Fig 7D and 7E). These data clearly suggest that HDJ2 inhibition is a promising strategy to sensitize cells to RNR inhibitors.

## Discussion

### Hsp70 co-chaperones regulate the cellular response to hydroxyurea

Hsp70 and Hsp90 bind a wide variety of client proteins, regulating many important signaling processes. Several studies have linked chaperone function to the DNA damage response and recently RNR activity [50–54]. Given that co-chaperones direct the activity and specificity of Hsp70 and Hsp90 it is logical that a subset of co-chaperones are responsible for supporting RNR activity. In this study, we identified 4 co-chaperones as being important for HU resistance; Ydj1, Scj1, Erj5 and Zuo1. Scj1 and Erj5 are ER-localized Hsp70 co-chaperones that bind to the ER-specific Hsp70 isoform Kar2. Kar2 and its co-chaperones are responsible for ER-folding and degradation of proteins (ERAD). Given their spatial separation from RNR components, these co-chaperones may support RNR activity indirectly or may have totally separate roles in DNA damage signaling. Zuo1 is a ribosome-associated chaperone that activates the ATPase activity of Ssb1 and Ssb2, making it likely that Zuo1 influences the transcription of RNR subunits or regulators. We hope to shed light on the role of these proteins in the DNA damage response in future studies.

### Ydj1 is required for both RNR subunit expression and stability

**Fig 8:**
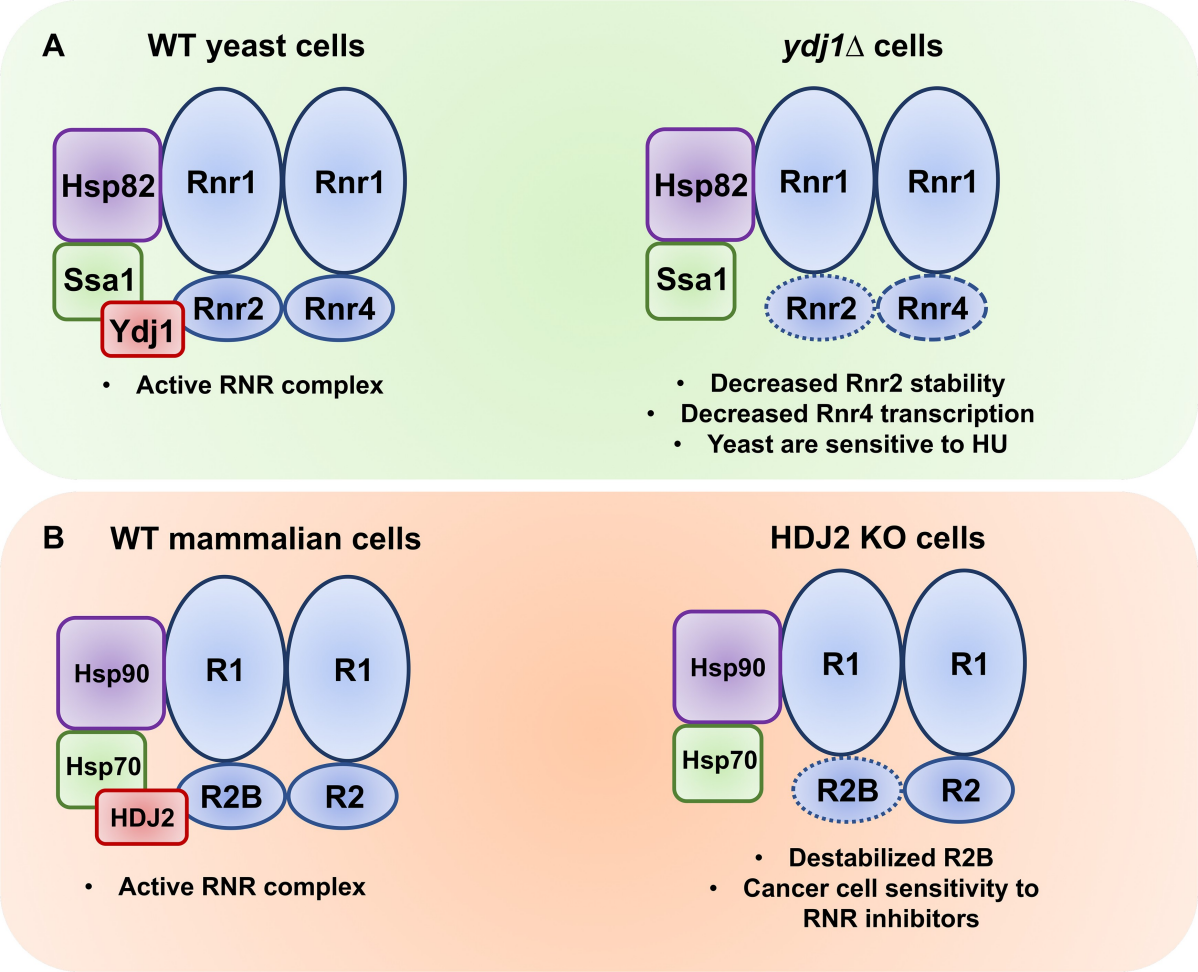
Ydj1/HDJ2 support RNR activity in yeast and mammalian cells. (A) In yeast, Ssa1, Hsp82 and Ydj1 bind and stabilize the RNR complex allowing dNTP synthesis required cell cycle progression and DNA repair. Loss of Ydj1 in yeast results in lowered Rnr2 levels (increased Rnr2 degradation) and Rnr4 (decreased *RNR4* transcription). (B) In mammalian cells, HSP70, HSP90 and HDJ2 bind and stabilize the RNR complex allowing dNTP synthesis required cell cycle progression and DNA repair. Loss of HDJ2 activity (through either CRISPR-mediated deletion or 116-9e) promotes lowering of R2B levels, sensitizing cells to RNR-inhibiting agents such as HU and triapine.

Ydj1 is a well-characterized Hsp40 responsible for mediating a large proportion of Hsp70 and Hsp90 effects in yeast. We show here that loss of Ydj1 results in lowered RNR expression via both transcription and protein stability. *RNR2* and *RNR*4 mRNA expression is decreased in *ydj1*Δ cells, although this effect seems to be relatively specific given that *RNR1* expression remains unchanged. It will be interesting to unravel the role of Ydj1 in global transcription in greater detail in future studies. In conjunction with decreased RNR expression, loss of Ydj1 significantly destabilizes Rnr2 (Fig 8). Again, although Ydj1 assists in the maturation and stability of numerous proteins in the cell [15], the effects here were specific. While Rnr2 half-life was lowered by loss of Ydj1, the half-lives of Rnr1 and Rnr4 remained unchanged. Rnr2 and Rnr4 hetero-dimerize *in vivo* and *in vitro* and they each support the others folding [27]. This may explain why overexpression of Rnr2 alone in *ydj1*Δ cells fails to suppress their HU-sensitive phenotype.

While the majority of co-chaperone function is mediated by interaction with chaperones such as Hsp90 and Hsp70, it is not unknown for co-chaperones to have chaperone-independent activities [55]. Ydj1 binds to Ssa1 via the conserved HPD region, and here we demonstrate that this interaction is required for Ydj1-mediated HU resistance. This is consistent with our previous studies identifying a role for Hsp90 and Hsp70 in regulating Rnr2 and Rnr4 [23]. These data suggest that either Ydj1 transports and transfers RNR components to Ssa1 or exists in a complex with RNR and Ssa1 to maintain RNR activity.

### Structural elements of Ydj1 required for HU resistance

Ydj1 is a well-characterized Hsp40 responsible for mediating a large proportion of Hsp70 and Hsp90 effects in yeast. Ydj1 exists as a dimer in yeast and contains several functional elements such as the J-domain, G/F domain, Zinc-finger-like domains and C-terminal domain (CTDII). It is interesting to note that the same C-terminal Ydj1 truncations that result in HU-sensitivity correspond with a previously observed loss of ability of cells to sustain high-temperature growth [16]. To tease apart the role of the Ydj1 N-terminus in HU resistance, we utilized chimeras of Ydj1 and its paralog Sis1. Although Sis1 and Ydj1 possess the ability to bind Ssa1, loss of Sis1 is lethal for yeast, whereas loss of Ydj1 is not [41]. Although individual replacement of either the N-terminal domain or G/F domain with the equivalent Sis1 domain had minimal impact on HU resistance, replacement of both domains simultaneously (SSY chimera) increased the cells sensitivity to HU to a level almost equivalent to *ydj1*Δ cells. In addition, while *ydj1*Δ cells are HU sensitive, they are not as sensitive as *rnr4*Δ cells and are viable (unlike *rnr2*Δ cells), suggesting that loss of Ydj1 does not result in total loss of RNR function. This is in agreement with our data that shows partial but not complete destabilization of RNR levels in *ydj1*Δ cells. One potential explanation for this is that the related Sis1 co-chaperone is partially functionally redundant with Ydj1 and can contribute to some degree in RNR activity, particularly given the data shown in Fig 2B.

It is compelling that Ydj1 farnesylation is required for HU resistance, as it is not required to bind all client proteins and only a small proportion of Ydj1 is farnesylated at any one time [14]. Farnesylation anchors Ydj1 to the exterior face of the endoplasmic reticulum (ER) where it functions to prevent the passage of aggregated proteins from mother to daughter cells during cell division [45]. While no previous connection between ER function and RNR activity has been identified, it is interesting to note in this study cells lacking of either of two ER specific co-chaperones Erdj5 and Scj1 are also HU sensitive. Although beyond the scope of this study, the interplay between ER co-chaperones and the DNA damage will be interesting to explore further.

### HDJ2 regulates RNR in cancer cells

Suggesting broad conservation of the yeast mechanism, we demonstrate here that human Ydj1 (HDJ2) and human RNR2 (R2B) physically interact and that this interaction is independent of HU treatment. This is interesting given our previous findings that RNR subunit interaction with both Hsp70 and Hsp90 increase under replicative stress [23, 24]. Our data suggest that both the Ydj1 (yeast) and HDJ2 (human) co-chaperones may recruit and transfer Rnr2/R2B to their respective chaperones (Ssa1/Hsp70) for folding, as loss of co-chaperone function or inability to form a productive chaperone-co-chaperone complex promotes Rnr2/R2B degradation (Fig 8).

### Inhibition of HDJ2 as a viable strategy to sensitize cancer cells to RNR inhibitors

Yeast lacking Ydj1 display a destabilized RNR complex and corresponding sensitivity to HU. In turn, we demonstrate that we can sensitize a cancer cells to HU and the more potent RNR inhibitor triapine by either CRISPR-mediated gene knockout of HDJ2 or by inhibiting HDJ2 with 116-9e. HU was the first small-molecule RNR approved in 1967. HU and other agents, including the nucleoside analog gemcitabine (Gemzar) and triapine, remain important agents in cancer chemotherapy. These agents are commonly combined with radiotherapy and/or genotoxic chemotherapy, which potentiate RNR inhibitors via exposing the requirement for dNTPs in DNA repair [25, 33]. It would be highly desirable to identify agents that can enhance the therapeutic benefit of RNR inhibitors without incurring additional toxicity.

Several studies have demonstrated the antitumor potential of small molecule inhibitors of chaperones, particularly Hsp90 [56]. Despite promising *in vitro* results several potent Hsp90 inhibitors such as 17-AAG have failed clinical trials due to solubility and toxicity issues [7]. Creating clinically-relevant Hsp70 inhibitors is also challenging, given that Hsp70 is responsible for both the stabilization and degradation of client proteins, many of which are required for cell viability in healthy cells. The ‘holy grail’ of chaperone-based translational research is how to modulate chaperone function in cells such that cancer cells are selectively targeted over healthy tissue.

An alternative strategy may be the targeting of specific co-chaperones, particularly Hsp40s. By replacing the dichlorobenzyl functionality of 115-7c (an Hsp70 activator) with a bulkier diphenyl group, Wisen et al. were able to create 116-9e, an inhibitor capable of specifically inhibiting the Hsp70-Hsp40 interaction in both yeast and mammalian cells [48]. We demonstrate for the first time that 116-9e has the ability to sensitize cancer cells to anticancer agents such as triapine and HU through destabilization of R2B. Interestingly, a recent study identified a novel Hsp40-binding molecule C86 as being capable of destabilizing the androgen receptor, a driver of metastasis in castration-resistant prostate cancer [22]. While we anticipate future studies to examine the dosing and timing required to optimize cancer cell inhibition, the results of both this study and that of [22] demonstrates the validity of destabilizing select client proteins in cancer through Hsp70 co-chaperone inhibition.

## Materials and methods

### Yeast Strains and growth conditions

Yeast cultures were grown in either YPD (1% yeast extract, 2% glucose, 2% peptone) or grown in SD (0.67% yeast nitrogen base without amino acids and carbohydrates, 2% glucose) supplemented with the appropriate nutrients to select for plasmids and tagged genes. *Escherichia coli* DH5α was used to propagate all plasmids. E. coli cells were cultured in Luria broth medium (1% Bacto tryptone, 0.5% Bacto yeast extract, 1% NaCl) and transformed to ampicillin resistance by standard methods. For tagging the genomic copy of *RNR1*, *RNR2* and *RNR4* with a GFP epitope at the carboxy-terminus, the pFA6a-GFP(S65T)-His3MX6 plasmid was used. A full table of yeast strains and plasmids that were used can be found in S1 Table. For serial dilutions, cells were grown to mid-log phase, 10-fold serially diluted and then plated onto appropriate media using a 48-pin replica-plating tool. Images of plates were taken after 3 days at 30°C. 200mM HU was used for serial dilutions and to stress yeast cells, a concentration established in [57].

### β-Galactosidase assays

For *RNR3-lacZ* fusion expression experiments, cells were grown overnight in SD-ura media at 30°C and then re-inoculated at OD_600_ of 0.2–0.4 and then grown for a further 4 hours. Cells were treated with 150 mM or 200 mM HU for 3 hours and then *RNR3-lacZ* fusion assays were carried out as described previously [58]. Briefly, protein was extracted through bead beating and protein was quantitated via Bradford assay. The b-Galactosidase reaction containing 50 μg of protein extract in 1 ml Z-Buffer (30) was initiated by addition of 200 μl ONPG (4 mg/ml) and incubated at 28°C until the appearance of a pale-yellow color was noted. The reaction was quenched via the addition of 500 μl Na_2_CO_3_ (1M) solution. The optical density of the reaction was measured at 420nm. β-Gal activity was calculated using ((OD_420_ x 1.7)/(0.0045 x protein x reaction time)), where protein is measured in mg, and time is in minutes. The mean and standard deviation from three independent transformants were calculated.

### Gal promoter shut off experiments

BY4742 WT or *ydj1*Δ cells transformed with either pGAL1-HA-Rnr1, 2 or 4 plasmid were grown to mid-log phase in YP Gal medium (1% yeast extract, 2% galactose, 2% peptone). Transcription of pGAL1-HA-Rnr1, 2 or 4 was shut off by addition of 2% glucose to cultures. Aliquots of cells were collected at 0, 2 and 4 hours after addition of glucose. Cell lysates from these samples were analyzed by Western Blotting for stability of RNR subunit (HA antibody) and loading control (GAPDH).

### Cyclohexamide experiments

For cycloheximide experiments, BY4742 WT and *ydj1*Δ cells expressing endogenous promoter GFP-tagged Rnr2 were grown to exponential phase in YPD media and then treated with 200 μg/ml cycloheximide for 6 hours to halt protein translation. Cell lysates were obtained and analyzed via SDS-PAGE/Western Blotting for GFP-Rnr2 (GFP antibody) and a GAPDH loading control (GAPDH antibody).

### Western blotting

Protein extracts were made as described (Kamada et al., 1995). 20 μg of protein was separated by 4%–12% NuPAGE SDS-PAGE (Thermo). Proteins were detected using the following antibodies; anti-HIS tag (QIAGEN #34670), anti-GFP (Roche #1814460), Anti-FLAG tag (Sigma, #F1365), anti-GAPDH (Thermo #MA5-15738), anti-Ydj1 (StressMarq #SMC-166D), anti-R2B (SCBT, #sc-376963), anti-HDJ2 (Thermo #MA512748).

Blots were imaged on a ChemiDoc MP imaging system (Bio-Rad). After treatment with SuperSignal West Pico Chemiluminescent Substrate (GE). Blots were stripped and re-probed with the relevant antibodies using Restore Western Blot Stripping Buffer (Thermo).

### Purification of FLAG-tagged Rnr2 from yeast

Cells transformed with control pRS313 plasmid or the pRS313 plasmid containing HIS-tagged Rnr2 were grown overnight in SD-HIS media, and then reinoculated into a larger culture of selectable media and grown to an OD_600_ of 0.800. The cells were then either unstressed or stressed with 200 mM HU for four hours. Cells were harvested and FLAG-tagged proteins were isolated as follows: Protein was extracted via bead beating in 500 μl binding buffer (50 mM Na-phosphate pH 8.0, 300 mM NaCl, 0.01% Tween-20). 200 μg of protein extract was incubated with 30 μl anti-Flag M2 magnetic beads (Sigma) at 4° C overnight. Anti-Flag M2 beads were collected by magnet then washed 5 times with 500 μl binding buffer. After the final wash, the buffer was aspirated and beads were incubated with 65 μl Elution buffer (binding buffer supplemented with 10 μg/ml 3X FLAG peptide (Apex Bio)) for 1 hour at 4° C, then beads were collected via magnet. The supernatant containing purified FLAG-Rnr2 was transferred to a fresh tube, 25 μl of 5x SDS-PAGE sample buffer was added and the sample was denatured for 5 min at 95° C. 20 μl of sample was analyzed by SDS-PAGE.

### Quantitation of yeast RNR subunit transcription

Quantitation of yeast RNR transcription was carried out as in [59]. Briefly, yeast cells were grown overnight in YPD media at 30°C, re-inoculated at OD_600_ of 0.2–0.4 and then grown for a further 4 hours. Cells were treated with 200 mM for 2 hours and total RNA was extracted from cells using a GeneJet RNA extraction kit. Total RNA (1 μg) was treated with 10 units of RNase-free DNase I (Thermo) for 30 min at 37°C to remove contaminating DNA. DNAse I activity was stopped by adding 1 μL of 50 mM EDTA and incubating at 65°C for 10 minutes. cDNA synthesis was carried out by iScript reverse transcriptase (BioRad) on aliquots of 1 μg RNA. The single-stranded cDNA products were used in qPCR on an ABI Fast 2000 real-time PCR detection system based on SYBR Green fluorescence. Sequences of oligo pairs (same as used in [59]) are listed in S1 Table. Signals of *RNR1*, *RNR2* and *RNR4* were normalized against that of *ACT1* in each strain and the resulting ratios in WT cells were defined as onefold.

### Mammalian cell culture and drug treatment

HEK293T cells were cultured in Dulbecco’s modified Eagle’s minimal essential medium (DMEM; Invitrogen, Carlsbad, CA, USA) supplemented with 10% fetal bovine serum (FBS; Invitrogen), 100 U/ml penicillin (Invitrogen) and 100 μg/ml streptomycin (Invitrogen). All cell lines were incubated at 37 C in a 5% CO2 containing atmosphere. For 116-9e treatment, HEK293 cells were treated with 116-9e (#E1036, Sigma) at 40 μM concentration and kept in incubator at 37°C and 5% CO_2_ for 72 hours. After 72h cells were washed with 1X PBS and total cell extracts were prepared using Mammalian Protein Extract Reagent (Thermo).

HAP1 cells and HDJ2 Knockout cells were obtained from Horizon Biosciences and were cultured in Iscove’s Modified Dulbecco’s Medium (IMDM) supplemented with 10% fetal bovine serum (FBS; Invitrogen), 100 U/ml penicillin (Invitrogen) and 100 μg/ml streptomycin (Invitrogen). For IC_50_ calculations, HAP1 cells and HDJ2 Knockout were seeded in triplicate in 96-well white bottom Nunc plates in growth media at 20% confluency 1 day prior to initiation of drug treatment. On Day 1 of treatment, cells were treated with a two-fold serial dilution of Hydroxyurea (400μM to 1.56 μM). After 72 h, cell viability was measured using Promega CellTiter-Glo cell viability assay on a Synergy H1 plate reader. Similarly, cells were treated with either DMSO (control) or 40 μM of 116-9e in combination with a ten-fold serial dilution of either HU (400μM to 1.56 μM) or triapine (250μM to 0.0005 μM). After 72 h, cell viability was measured using Promega CellTiter-Glo cell viability assay on a Synergy H1 plate reader.

### Purification of HIS-tagged proteins from mammalian cells

HEK293T cells were either un-transfected or transfected with plasmids for expression of HIS-tagged proteins using Lipofectamine 3000 (Thermo). After 48 hours, the cells were washed with 1XPBS and total cell extract was prepared from the cells using M-PER (Thermo) containing EDTA-free protease and phosphatase inhibitor cocktail (Thermo) according to the manufacturer's recommended protocol. Protein was quantitated using the Bradford Assay. His-tagged proteins were purified as follows: 200 μg of cell lysate was incubated with 30 μl of His-Tag Dynabeads (Invitrogen) with gentle agitation for 20 minutes at 4° C. Dynabeads were collected by magnet then washed 5 times with 500 μl Binding/Wash buffer. After final wash, buffer was aspirated and beads were incubated with 65 μl Elution buffer (300 mM imidazole, 50 mM Na-phosphate pH 8.0, 300 mM NaCl, 0.01% Tween-20) for 20 min, then beads were collected via magnet. The supernatant containing the purified HIS-tagged protein complex was transferred to a fresh tube, 15 μl of 5x SDS-PAGE sample buffer was added and the sample was denatured for 5 min at 95° C. 20 μl of sample was analyzed by SDS-PAGE and Western Blotting.

### Combination index (CI) calculations

HAP1 cells were seeded in triplicate in 96-well white bottom Nunc plates in growth media at 20% confluency 1 day prior to initiation of drug treatment. On Day 1 of treatment, cells were treated with DMSO (control) and serial dilutions of Hydroxyurea and 116-9e. After 72 h, cell viability was measured using Promega CellTiter-Glo cell viability assay on a 96-well plate reader. The combination index was calculated using the Chou-Talalay method using CompuSyn software [60].

## Supporting information

S1 Table Yeast strains and plasmids used in this study. A table of yeast strain and plasmids used in the study are described, along with their original source, genotypes and selectable markers.
(PDF)

S1 Fig A selection of yeast co-chaperone mutants are HU sensitive. WT BY4742 or BY4742 cells lacking Rnr4 or 28 co-chaperone proteins were grown overnight to saturation and serial 10-fold dilutions were plated by pin plating from 96-well plates onto YPD alone or YPD containing 200 mM HU. Plates were imaged after 3 days. HU-sensitive co-chaperone mutant strains are highlighted in red.
(TIF)

S2 Fig Relative growth defects of BY4742 *ydj1*Δ and JJ160. BY4742 WT, BY4742 *ydj1***Δ** and JJ160 cells were grown overnight to saturation and serial 10-fold dilutions were plated by pin plating from 96-well plates onto YPD alone or YPD containing either 150mM or 200 mM HU. Plates were imaged after 3 days.
(TIF)

S3 Fig Genomic tagging of RNR subunits in BY4742 *ydj1*Δ does not enhance the *ydj1*Δ growth defect. BY4742 WT *and* BY4742 *ydj1***Δ** cells were grown overnight to saturation and serial 10-fold dilutions were plated by pin plating from 96-well plates onto YPD. Plates were imaged after 3 days.
(TIF)

S4 Fig Overexpression of Rnr2 using a multicopy, strong promoter system. (A) BY4742 WT cells expressing either endogenously tagged Rnr2-GFP or BY4742 WT transformed with plasmid expressing Rnr2-GFP from a constitutively high *MET25* promoter. Cell extracts were obtained, resolved on SDS-PAGE gels and analyzed by immunoblotting with anti-GFP and GAPDH antibodies. (B) Even when expressed from a constitutive promoter, Rnr2 levels are lower in *ydj1***Δ** cells. WT and *ydj1***Δ** cells were transformed with a multicopy plasmid expressing Rnr2-GFP from the constitutive *MET25* promoter. Extracts were obtained as above, resolved on SDS-PAGE gels and analyzed by immunoblotting with anti-GFP and GAPDH antibodies.
(TIF)

## References

[1] Craig EA, Marszalek J. How Do J-Proteins Get Hsp70 to Do So Many Different Things? Trends Biochem Sci. 2017;42(5):355–68. Epub 2017/03/21. 10.1016/j.tibs.2017.02.007 ; PubMed Central PMCID: PMCPMC5409888. 28314505 10.1016/j.tibs.2017.02.007 PMC5409888

[2] Kim YE, Hipp MS, Bracher A, Hayer-Hartl M, Hartl FU. Molecular chaperone functions in protein folding and proteostasis. Annual review of biochemistry. 2013;82:323–55. Epub 2013/06/12. 10.1146/annurev-biochem-060208-092442 . 23746257 10.1146/annurev-biochem-060208-092442

[3] Nillegoda NB, Wentink AS, Bukau B. Protein Disaggregation in Multicellular Organisms. Trends Biochem Sci. 2018;43(4):285–300. Epub 2018/03/05. 10.1016/j.tibs.2018.02.003 . 29501325 10.1016/j.tibs.2018.02.003

[4] Lianos GD, Alexiou GA, Mangano A, Mangano A, Rausei S, Boni L, et al The role of heat shock proteins in cancer. Cancer letters. 2015;360(2):114–8. Epub 2015/02/28. 10.1016/j.canlet.2015.02.026 . 25721081 10.1016/j.canlet.2015.02.026

[5] Shevtsov M, Huile G, Multhoff G. Membrane heat shock protein 70: a theranostic target for cancer therapy. Philosophical transactions of the Royal Society of London Series B, Biological sciences. 2018;373(1738). Epub 2017/12/06. 10.1098/rstb.2016.0526 ; PubMed Central PMCID: PMCPMC5717526. 29203711 10.1098/rstb.2016.0526 PMC5717526

[6] Rohde M, Daugaard M, Jensen MH, Helin K, Nylandsted J, Jaattela M. Members of the heat-shock protein 70 family promote cancer cell growth by distinct mechanisms. Genes Dev. 2005;19(5):570–82. 10.1101/gad.305405 ; PubMed Central PMCID: PMC551577. 15741319 10.1101/gad.305405 PMC551577

[7] Erlichman C. Tanespimycin: the opportunities and challenges of targeting heat shock protein 90. Expert Opin Investig Drugs. 2009;18(6):861–8. Epub 2009/05/27. 10.1517/13543780902953699 . 19466875 10.1517/13543780902953699

[8] Kampinga HH, Craig EA. The HSP70 chaperone machinery: J proteins as drivers of functional specificity. Nat Rev Mol Cell Biol. 2011;11(8):579–92. Epub 2010/07/24. nrm2941 [pii] 10.1038/nrm2941 ; PubMed Central PMCID: PMC3003299. 20651708 10.1038/nrm2941 PMC3003299

[9] Walsh P, Bursac D, Law YC, Cyr D, Lithgow T. The J-protein family: modulating protein assembly, disassembly and translocation. EMBO Rep. 2004;5(6):567–71. Epub 2004/06/02. 10.1038/sj.embor.7400172 ; PubMed Central PMCID: PMCPMC1299080. 15170475 10.1038/sj.embor.7400172 PMC1299080

[10] Luke MM, Sutton A, Arndt KT. Characterization of SIS1, a Saccharomyces cerevisiae homologue of bacterial dnaJ proteins. J Cell Biol. 1991;114(4):623–38. Epub 1991/08/01. ; PubMed Central PMCID: PMC2289895. 1714460 10.1083/jcb.114.4.623 PMC2289895

[11] Caplan AJ, Douglas MG. Characterization of YDJ1: a yeast homologue of the bacterial dnaJ protein. J Cell Biol. 1991;114(4):609–21. Epub 1991/08/01. ; PubMed Central PMCID: PMC2289889. 1869583 10.1083/jcb.114.4.609 PMC2289889

[12] Kirkland PA, Reidy M, Masison DC. Functions of yeast Hsp40 chaperone Sis1p dispensable for prion propagation but important for prion curing and protection from prion toxicity. Genetics. 2011;188(3):565–77. Epub 2011/05/11. genetics.111.129460 [pii] 10.1534/genetics.111.129460 ; PubMed Central PMCID: PMC3176549. 21555396 10.1534/genetics.111.129460 PMC3176549

[13] Escusa-Toret S, Vonk WI, Frydman J. Spatial sequestration of misfolded proteins by a dynamic chaperone pathway enhances cellular fitness during stress. Nat Cell Biol. 2013;15(10):1231–43. Epub 2013/09/17. 10.1038/ncb2838 ; PubMed Central PMCID: PMCPMC4121856. 24036477 10.1038/ncb2838 PMC4121856

[14] Flom GA, Lemieszek M, Fortunato EA, Johnson JL. Farnesylation of Ydj1 is required for in vivo interaction with Hsp90 client proteins. Mol Biol Cell. 2008;19(12):5249–58. Epub 2008/10/03. E08-04-0435 [pii] 10.1091/mbc.E08-04-0435 ; PubMed Central PMCID: PMC2592663. 18829866 10.1091/mbc.E08-04-0435 PMC2592663

[15] Mandal AK, Nillegoda NB, Chen JA, Caplan AJ. Ydj1 protects nascent protein kinases from degradation and controls the rate of their maturation. Mol Cell Biol. 2008;28(13):4434–44. Epub 2008/04/30. MCB.00543-08 [pii] 10.1128/MCB.00543-08 ; PubMed Central PMCID: PMC2447146. 18443039 10.1128/MCB.00543-08 PMC2447146

[16] Johnson JL, Craig EA. A role for the Hsp40 Ydj1 in repression of basal steroid receptor activity in yeast. Mol Cell Biol. 2000;20(9):3027–36. Epub 2000/04/11. ; PubMed Central PMCID: PMC85575. 10757787 10.1128/mcb.20.9.3027-3036.2000 PMC85575

[17] Moriel-Carretero M, Tous C, Aguilera A. Control of the function of the transcription and repair factor TFIIH by the action of the cochaperone Ydj1. Proc Natl Acad Sci U S A. 2011;108(37):15300–5. Epub 2011/08/31. 1107425108 [pii] 10.1073/pnas.1107425108 ; PubMed Central PMCID: PMC3174667. 21876155 10.1073/pnas.1107425108 PMC3174667

[18] Stark JL, Mehla K, Chaika N, Acton TB, Xiao R, Singh PK, et al Structure and function of human DnaJ homologue subfamily a member 1 (DNAJA1) and its relationship to pancreatic cancer. Biochemistry. 2014;53(8):1360–72. Epub 2014/02/12. 10.1021/bi401329a ; PubMed Central PMCID: PMCPMC3985919. 24512202 10.1021/bi401329a PMC3985919

[19] Mitra A, Shevde LA, Samant RS. Multi-faceted role of HSP40 in cancer. Clin Exp Metastasis. 2009;26(6):559–67. Epub 2009/04/03. 10.1007/s10585-009-9255-x . 19340594 10.1007/s10585-009-9255-x

[20] Sterrenberg JN, Blatch GL, Edkins AL. Human DNAJ in cancer and stem cells. Cancer letters. 2011;312(2):129–42. Epub 2011/09/20. 10.1016/j.canlet.2011.08.019 . 21925790 10.1016/j.canlet.2011.08.019

[21] Assimon VA, Gillies AT, Rauch JN, Gestwicki JE. Hsp70 protein complexes as drug targets. Curr Pharm Des. 2013;19(3):404–17. Epub 2012/08/28. ; PubMed Central PMCID: PMC3593251. 22920901 10.2174/138161213804143699 PMC3593251

[22] Moses MA, Kim YS, Rivera-Marquez GM, Oshima N, Watson MJ, Beebe KE, et al Targeting the Hsp40/Hsp70 Chaperone Axis as a Novel Strategy to Treat Castration-Resistant Prostate Cancer. Cancer Res. 2018;78(14):4022–35. Epub 2018/05/17. 10.1158/0008-5472.CAN-17-3728 ; PubMed Central PMCID: PMCPMC6050126. 29764864 10.1158/0008-5472.CAN-17-3728 PMC6050126

[23] Truman AW, Kristjansdottir K, Wolfgeher D, Ricco N, Mayampurath A, Volchenboum SL, et al Quantitative proteomics of the yeast Hsp70/Hsp90 interactomes during DNA damage reveal chaperone-dependent regulation of ribonucleotide reductase. Journal of proteomics. 2015;112:285–300. Epub 2014/12/03. 10.1016/j.jprot.2014.09.028 ; PubMed Central PMCID: PMCPMC4485990. 25452130 10.1016/j.jprot.2014.09.028 PMC4485990

[24] Truman AW, Kristjansdottir K, Wolfgeher D, Ricco N, Mayampurath A, Volchenboum SL, et al The quantitative changes in the yeast Hsp70 and Hsp90 interactomes upon DNA damage. Data Brief. 2015;2:12–5. Epub 2015/07/29. 10.1016/j.dib.2014.10.006 ; PubMed Central PMCID: PMCPMC4459869. 26217697 10.1016/j.dib.2014.10.006 PMC4459869

[25] Sanvisens N, de Llanos R, Puig S. Function and regulation of yeast ribonucleotide reductase: cell cycle, genotoxic stress, and iron bioavailability. Biomed J. 2013;36(2):51–8. Epub 2013/05/07. 10.4103/2319-4170.110398 . 23644233 10.4103/2319-4170.110398

[26] Cotruvo JA, Stubbe J. Escherichia coli class Ib ribonucleotide reductase contains a dimanganese(III)-tyrosyl radical cofactor in vivo. Biochemistry. 2011;50(10):1672–81. Epub 2011/01/22. 10.1021/bi101881d ; PubMed Central PMCID: PMCPMC3076206. 21250660 10.1021/bi101881d PMC3076206

[27] Perlstein DL, Ge J, Ortigosa AD, Robblee JH, Zhang Z, Huang M, et al The active form of the Saccharomyces cerevisiae ribonucleotide reductase small subunit is a heterodimer in vitro and in vivo. Biochemistry. 2005;44(46):15366–77. Epub 2005/11/16. 10.1021/bi051616+ ; PubMed Central PMCID: PMCPMC4669231. 16285741 10.1021/bi051616+ PMC4669231

[28] Kolberg M, Strand KR, Graff P, Andersson KK. Structure, function, and mechanism of ribonucleotide reductases. Biochim Biophys Acta. 2004;1699(1–2):1–34. Epub 2004/05/26. 10.1016/j.bbapap.2004.02.007 . 15158709 10.1016/j.bbapap.2004.02.007

[29] Chabes A, Domkin V, Larsson G, Liu A, Graslund A, Wijmenga S, et al Yeast ribonucleotide reductase has a heterodimeric iron-radical-containing subunit. Proc Natl Acad Sci U S A. 2000;97(6):2474–9. ; PubMed Central PMCID: PMC15953. 10716984 10.1073/pnas.97.6.2474 PMC15953

[30] Elledge SJ, Davis RW. Two genes differentially regulated in the cell cycle and by DNA-damaging agents encode alternative regulatory subunits of ribonucleotide reductase. Genes Dev. 1990;4(5):740–51. Epub 1990/05/01. . 2199320 10.1101/gad.4.5.740

[31] Huang M, Zhou Z, Elledge SJ. The DNA replication and damage checkpoint pathways induce transcription by inhibition of the Crt1 repressor. Cell. 1998;94(5):595–605. Epub 1998/09/19. . 9741624 10.1016/s0092-8674(00)81601-3

[32] Jia X, Xiao W. Compromised DNA repair enhances sensitivity of the yeast RNR3-lacZ genotoxicity testing system. Toxicol Sci. 2003;75(1):82–8. Epub 2003/06/14. 10.1093/toxsci/kfg158 . 12805645 10.1093/toxsci/kfg158

[33] Cerqueira NM, Fernandes PA, Ramos MJ. Ribonucleotide reductase: a critical enzyme for cancer chemotherapy and antiviral agents. Recent Pat Anticancer Drug Discov. 2007;2(1):11–29. Epub 2008/01/29. . 18221051 10.2174/157489207779561408

[34] Cerqueira NM, Pereira S, Fernandes PA, Ramos MJ. Overview of ribonucleotide reductase inhibitors: an appealing target in anti-tumour therapy. Current medicinal chemistry. 2005;12(11):1283–94. Epub 2005/06/25. . 15974997 10.2174/0929867054020981

[35] Hsu HW, Wall NR, Hsueh CT, Kim S, Ferris RL, Chen CS, et al Combination antiangiogenic therapy and radiation in head and neck cancers. Oral Oncol. 2014;50(1):19–26. Epub 2013/11/26. 10.1016/j.oraloncology.2013.10.003 . 24269532 10.1016/j.oraloncology.2013.10.003

[36] Loehrer PJ Sr., Feng Y, Cardenes H, Wagner L, Brell JM, Cella D, et al Gemcitabine alone versus gemcitabine plus radiotherapy in patients with locally advanced pancreatic cancer: an Eastern Cooperative Oncology Group trial. J Clin Oncol. 2011;29(31):4105–12. Epub 2011/10/05. 10.1200/JCO.2011.34.8904 ; PubMed Central PMCID: PMCPMC3525836. 21969502 10.1200/JCO.2011.34.8904 PMC3525836

[37] Mornex F, Girard N. Gemcitabine and radiation therapy in non-small cell lung cancer: state of the art. Ann Oncol. 2006;17(12):1743–7. Epub 2006/06/13. 10.1093/annonc/mdl117 . 16766586 10.1093/annonc/mdl117

[38] Ghadban T, Dibbern JL, Reeh M, Miro JT, Tsui TY, Wellner U, et al HSP90 is a promising target in gemcitabine and 5-fluorouracil resistant pancreatic cancer. Apoptosis. 2017;22(3):369–80. Epub 2016/11/24. 10.1007/s10495-016-1332-4 . 27878398 10.1007/s10495-016-1332-4

[39] Li J, Qian X, Sha B. Heat shock protein 40: structural studies and their functional implications. Protein Pept Lett. 2009;16(6):606–12. Epub 2009/06/13. ; PubMed Central PMCID: PMCPMC2755554. 19519518 10.2174/092986609788490159 PMC2755554

[40] Johnson JL, Craig EA. An essential role for the substrate-binding region of Hsp40s in Saccharomyces cerevisiae. J Cell Biol. 2001;152(4):851–6. Epub 2001/03/27. ; PubMed Central PMCID: PMC2195774. 11266475 10.1083/jcb.152.4.851 PMC2195774

[41] Yan W, Craig EA. The glycine-phenylalanine-rich region determines the specificity of the yeast Hsp40 Sis1. Mol Cell Biol. 1999;19(11):7751–8. Epub 1999/10/19. ; PubMed Central PMCID: PMC84827. 10523664 10.1128/mcb.19.11.7751 PMC84827

[42] Reidy M, Sharma R, Shastry S, Roberts BL, Albino-Flores I, Wickner S, et al Hsp40s specify functions of Hsp104 and Hsp90 protein chaperone machines. PLoS Genet. 2014;10(10):e1004720 Epub 2014/10/21. 10.1371/journal.pgen.1004720 ; PubMed Central PMCID: PMCPMC4199505. 25329162 10.1371/journal.pgen.1004720 PMC4199505

[43] Borges JC, Seraphim TV, Mokry DZ, Almeida FC, Cyr DM, Ramos CH. Identification of regions involved in substrate binding and dimer stabilization within the central domains of yeast Hsp40 Sis1. PLoS One. 2012;7(12):e50927 Epub 2012/12/12. 10.1371/journal.pone.0050927 ; PubMed Central PMCID: PMCPMC3515540. 23227221 10.1371/journal.pone.0050927 PMC3515540

[44] Caplan AJ, Tsai J, Casey PJ, Douglas MG. Farnesylation of YDJ1p is required for function at elevated growth temperatures in Saccharomyces cerevisiae. J Biol Chem. 1992;267(26):18890–5. Epub 1992/09/15. . 1527016

[45] Saarikangas J, Caudron F, Prasad R, Moreno DF, Bolognesi A, Aldea M, et al Compartmentalization of ER-Bound Chaperone Confines Protein Deposit Formation to the Aging Yeast Cell. Curr Biol. 2017;27(6):773–83. Epub 2017/03/07. 10.1016/j.cub.2017.01.069 . 28262489 10.1016/j.cub.2017.01.069

[46] Fan CY, Lee S, Cyr DM. Mechanisms for regulation of Hsp70 function by Hsp40. Cell Stress Chaperones. 2003;8(4):309–16. Epub 2004/04/30. ; PubMed Central PMCID: PMCPMC514902. 15115283 10.1379/1466-1268(2003)008<0309:mfrohf>2.0.co;2 PMC514902

[47] Tsai J, Douglas MG. A conserved HPD sequence of the J-domain is necessary for YDJ1 stimulation of Hsp70 ATPase activity at a site distinct from substrate binding. J Biol Chem. 1996;271(16):9347–54. Epub 1996/04/19. . 8621599 10.1074/jbc.271.16.9347

[48] Wisen S, Bertelsen EB, Thompson AD, Patury S, Ung P, Chang L, et al Binding of a small molecule at a protein-protein interface regulates the chaperone activity of hsp70-hsp40. ACS chemical biology. 2010;5(6):611–22. Epub 2010/05/21. 10.1021/cb1000422 ; PubMed Central PMCID: PMC2950966. 20481474 10.1021/cb1000422 PMC2950966

[49] Balchin D, Hayer-Hartl M, Hartl FU. In vivo aspects of protein folding and quality control. Science. 2016;353(6294):aac4354 Epub 2016/07/02. 10.1126/science.aac4354 . 27365453 10.1126/science.aac4354

[50] Arlander SJ, Eapen AK, Vroman BT, McDonald RJ, Toft DO, Karnitz LM. Hsp90 inhibition depletes Chk1 and sensitizes tumor cells to replication stress. J Biol Chem. 2003;278(52):52572–7. Epub 2003/10/23. 10.1074/jbc.M309054200 . 14570880 10.1074/jbc.M309054200

[51] Quanz M, Herbette A, Sayarath M, de Koning L, Dubois T, Sun JS, et al Heat shock protein 90alpha (Hsp90alpha) is phosphorylated in response to DNA damage and accumulates in repair foci. J Biol Chem. 2012;287(12):8803–15. Epub 2012/01/25. 10.1074/jbc.M111.320887 ; PubMed Central PMCID: PMC3308794. 22270370 10.1074/jbc.M111.320887 PMC3308794

[52] Solier S, Kohn KW, Scroggins B, Xu W, Trepel J, Neckers L, et al Heat shock protein 90alpha (HSP90alpha), a substrate and chaperone of DNA-PK necessary for the apoptotic response. Proc Natl Acad Sci U S A. 2012;109(32):12866–72. Epub 2012/07/04. 10.1073/pnas.1203617109 ; PubMed Central PMCID: PMCPMC3420188. 22753480 10.1073/pnas.1203617109 PMC3420188

[53] Pennisi R, Ascenzi P, di Masi A. Hsp90: A New Player in DNA Repair? Biomolecules. 2015;5(4):2589–618. Epub 2015/10/27. 10.3390/biom5042589 ; PubMed Central PMCID: PMCPMC4693249. 26501335 10.3390/biom5042589 PMC4693249

[54] Li QQ, Hao JJ, Zhang Z, Krane LS, Hammerich KH, Sanford T, et al Proteomic analysis of proteome and histone post-translational modifications in heat shock protein 90 inhibition-mediated bladder cancer therapeutics. Sci Rep. 2017;7(1):201 Epub 2017/03/17. 10.1038/s41598-017-00143-6 ; PubMed Central PMCID: PMCPMC5427839. 28298630 10.1038/s41598-017-00143-6 PMC5427839

[55] Echtenkamp FJ, Zelin E, Oxelmark E, Woo JI, Andrews BJ, Garabedian M, et al Global functional map of the p23 molecular chaperone reveals an extensive cellular network. Mol Cell. 2011;43(2):229–41. Epub 2011/07/23. 10.1016/j.molcel.2011.05.029 ; PubMed Central PMCID: PMC3155841. 21777812 10.1016/j.molcel.2011.05.029 PMC3155841

[56] Trepel J, Mollapour M, Giaccone G, Neckers L. Targeting the dynamic HSP90 complex in cancer. Nature reviews Cancer. 2010;10(8):537–49. 10.1038/nrc2887 . 20651736 10.1038/nrc2887 PMC6778733

[57] Tkach JM, Yimit A, Lee AY, Riffle M, Costanzo M, Jaschob D, et al Dissecting DNA damage response pathways by analysing protein localization and abundance changes during DNA replication stress. Nat Cell Biol. 2012;14(9):966–76. Epub 2012/07/31. 10.1038/ncb2549 ; PubMed Central PMCID: PMC3434236. 22842922 10.1038/ncb2549 PMC3434236

[58] Truman AW, Millson SH, Nuttall JM, Mollapour M, Prodromou C, Piper PW. In the yeast heat shock response, Hsf1-directed induction of Hsp90 facilitates the activation of the Slt2 (Mpk1) mitogen-activated protein kinase required for cell integrity. Eukaryot Cell. 2007;6(4):744–52. Epub 2007/02/13. EC.00009-07 [pii] 10.1128/EC.00009-07 ; PubMed Central PMCID: PMC1865661. 17293484 10.1128/EC.00009-07 PMC1865661

[59] Zhang Y, Li H, Zhang C, An X, Liu L, Stubbe J, et al Conserved electron donor complex Dre2-Tah18 is required for ribonucleotide reductase metallocofactor assembly and DNA synthesis. Proc Natl Acad Sci U S A. 2014;111(17):E1695–704. Epub 2014/04/16. 10.1073/pnas.1405204111 ; PubMed Central PMCID: PMCPMC4035922. 24733891 10.1073/pnas.1405204111 PMC4035922

[60] Chou TC. Drug combination studies and their synergy quantification using the Chou-Talalay method. Cancer Res. 2010;70(2):440–6. Epub 2010/01/14. 10.1158/0008-5472.CAN-09-1947 . 20068163 10.1158/0008-5472.CAN-09-1947

