# Supplementary material for: The Hsp70 co-chaperone Ydj1/HDJ2 regulates ribonucleotide reductase activity
Source: PLoS Genet. 2018 Nov 19;14(11):e1007462. doi: 10.1371/journal.pgen.1007462 (PMC6277125; doi:10.1371/journal.pgen.1007462)
Supplement: S1 Table — A table of yeast strain and plasmids used in the study are described, along with their original source, genotypes and selectable markers. (PDF) [file pgen.1007462.s001.pdf]

Yeast strains used in this study

Strain	Genotype	Source
ATY8	MATα S288c (BY4742) *his3Δ1 leu2Δ0 lys2Δ0 ura3Δ0*	Euroscarf
ATY9	MATα S288c (BY4742) *rnr4Δ::KanMX4*	Euroscarf
ATY10	MATα S288c (BY4742) *erj5Δ::KanMX4*	Euroscarf
ATY11	MATα S288c (BY4742) *aha1Δ::KanMX4*	Euroscarf
ATY12	MATα S288c (BY4742) *swa2Δ::KanMX4*	Euroscarf
ATY13	MATα S288c (BY4742) *ppt1Δ::KanMX4*	Euroscarf
ATY14	MATα S288c (BY4742) *cpr6Δ::KanMX4*	Euroscarf
ATY15	MATα S288c (BY4742) *jjj2Δ::KanMX4*	Euroscarf
ATY16	MATα S288c (BY4742) *lhs1Δ::KanMX4*	Euroscarf
ATY17	MATα S288c (BY4742) *sba1Δ::KanMX4*	Euroscarf
ATY18	MATα S288c (BY4742) *sse1Δ::KanMX4*	Euroscarf
ATY19	MATα S288c (BY4742) *sil1Δ::KanMX4*	Euroscarf
ATY20	MATα S288c (BY4742) *sti1Δ::KanMX4*	Euroscarf
ATY21	MATα S288c (BY4742) *mdj2Δ::KanMX4*	Euroscarf
ATY22	MATα S288c (BY4742) *xdj1Δ::KanMX4*	Euroscarf
ATY23	MATα S288c (BY4742) *caj1Δ::KanMX4*	Euroscarf
ATY24	MATα S288c (BY4742) *hij1Δ::KanMX4*	Euroscarf
ATY25	MATα S288c (BY4742) *scj1Δ::KanMX4*	Euroscarf
ATY26	MATα S288c (BY4742) *snl1Δ::KanMX4*	Euroscarf
ATY27	MATα S288c (BY4742) *apj1Δ::KanMX4*	Euroscarf
ATY28	MATα S288c (BY4742) *ydj1Δ::KanMX4*	Euroscarf
ATY29	MATα S288c (BY4742) *cpr7Δ::KanMX4*	Euroscarf
ATY30	MATα S288c (BY4742) *jj3Δ::KanMX4*	Euroscarf
ATY31	MATα S288c (BY4742) *jem1Δ::KanMX4*	Euroscarf
ATY32	MATα S288c (BY4742) *sec63Δ::KanMX4*	Euroscarf
ATY33	MATα S288c (BY4742) *zuo1Δ::KanMX4*	Euroscarf
ATY34	MATα S288c (BY4742) *tah1Δ::KanMX4*	Euroscarf
ATY35	MATα S288c (BY4742) *sse2Δ::KanMX4*	Euroscarf
ATY36	MATα S288c (BY4742) *jj1Δ::KanMX4*	Euroscarf
ATY37	MATα S288c (BY4742) *fes1Δ::KanMX4*	Euroscarf
ATY222	MATα S288c (BY4742):: *RNR1-GFP-HIS3MX6*	This study
ATY227	MATα S288c (BY4742):: *ydj1Δ::kanMX4 RNR1-GFP-HIS3MX6*	This study
ATY229	MATα S288c (BY4742):: *RNR2-GFP-HIS3MX6*	This study
ATY233	MATα S288c (BY4742) *ydj1Δ::KanMX4:: RNR2-GFP-His3MX6*	This study
ATY236	MATα S288c (BY4742):: *RNR4-GFP-HIS3MX6*	This study
ATY240	MATα S288c (BY4742) *ydj1Δ::KanMX4:: RNR4-GFP-HIS3MX6*	This study
ATY264	JJ160 (a *trp-1 ura3-1 leu2-3,112 his3-11,15 ade2-1 can1-100 GAL2Δ met2-Δ1 lys2-Δ2 ydj1::HIS3) pRS316-YDJ1*	EA Craig
ATY265	JJ160 *pRS315-YDJ1*	EA Craig
ATY267	JJ160 *pRS315-YDJ1(1-134)*	EA Craig
ATY269	JJ160 *pRS315-YDJ1(1-206)*	EA Craig
ATY270	JJ160 *pRS315-YDJ1(1-363)*	EA Craig
ATY272	JJ160 *pRS315-YDJ1(G153R)*	EA Craig
ATY273	JJ160 *pRS315-YDJ1(G315D)*	EA Craig
ATY274	JJ160 *pRS315-YDJ1(C406S)*	EA Craig
ATY275	JJ160 *pRS315*	This study
ATY280	JJ160 *pMR267* (Ydj1-cMyc)	This study
ATY281	JJ160 *pMR267* (Ydj1-D36N-cMyc)	This study
ATY282	JJ160 *pMR275* (SYY c-Myc)	This study
ATY283	JJ160 *pMR277* (YSY c-Myc)	This study
ATY284	JJ160 *pMR278* (SSY c-Myc)	This study
ATY285	JJ160 *pRS314*	This study
ATY362	JJ160 *::RNR2-GFP-KANMX4 pMR267* Ydj1-cMyc	This study
ATY363	JJ160 *::RNR2-GFP-KANMX4 pMR267* Ydj1(D36N)-cMyc	This study

Plamsids used in this study

Plasmid	Description	Referenece/Source
ATP79	pCMV3.1 HIS-FLAG HSC70	This study
ATP399	pCMV3.1 HIS-R2B	This study
ATP400	pUG36-RNR2-GFP	This study
ATP433	pUG36	J. H. Hegemann
ATP573	pMR278 (SSY c-Myc)	D. Masison
ATP574	pMR277 (YSY c-Myc)	D. Masison
ATP575	pMR275 (SYY c-Myc)	D. Masison
ATP576	pMR267 (Ydj1-D36N-cMyc)	D. Masison
ATP578	pMR267 (Ydj1-cMyc)	E A Craig
ATP580	pRS315-YDJ1(1-134)	E A Craig
ATP582	pRS315-YDJ1(1-206)	E A Craig
ATP584	pRS315-YDJ1(1-363)	E A Craig
ATP586	pRS315-YDJ1(G153R)	E A Craig
ATP587	pRS315-YDJ1(G315D)	E A Craig
ATP588	pRS315-YDJ1(C406S)	E A Craig
ATP-590	pRS413-*RNR2* promoter-FLAG-RNR2	M Huang
ATP592	*RNR3* promoter-lacZ	W. Xiao
ATP716	pBG1085-*GAL1* promoter-ZZ-HA-RNR1	Dharmacon
ATP717	pBG1085-GAL1 promoter-ZZ-HA-RNR2	Dharmacon
ATP718	pBG1085-GAL1 promoter-ZZ-HA-RNR4	Dharmacon
ATP-723	pFA6a-GFP(S65T)-KANMX6	J. Pringle
ATP-724	pFA6a-GFP(S65T)-HIS3MX6	J. Pringle

Table of primers used for RT-qPCR

Name	Sequence
*RNR1*_F	5′-GTGTTCAAGGTCTCGCTGAC-3′
*RNR1*_R	5′-CGTATGGACCGTCCTTCTGA-3′
*RNR2*_F	5′-CCTAAAGAGACCCCTTCCAAAG-3′
*RNR2*_R	5′-GCCTTGTGATTTTCAGCGTC-3′
*RNR3*_F	5′-GCCTCCGCTGCTATTCAA-3′
*RNR3*_R	5′- CAGATGCCGCCTTTTGTT-3′
*RNR4*_F	5′-CATAAGGCTGCTTTCATCGAG-3′
*RNR4*_R	5′-CTGTTGGCCATTGCTAAACC-3′
*ACT1*_F	5′-GTATGTGTAAAGCCGGTTTTG-3′
*ACT1*_R	5′-CATGATACCTTGGTGTCTTGG-3′
