# Supplementary figures and images for: The Hsp70 co-chaperone Ydj1/HDJ2 regulates ribonucleotide reductase activity
Source: PLoS Genet. 2018 Nov 19;14(11):e1007462. doi: 10.1371/journal.pgen.1007462 (PMC6277125; doi:10.1371/journal.pgen.1007462)

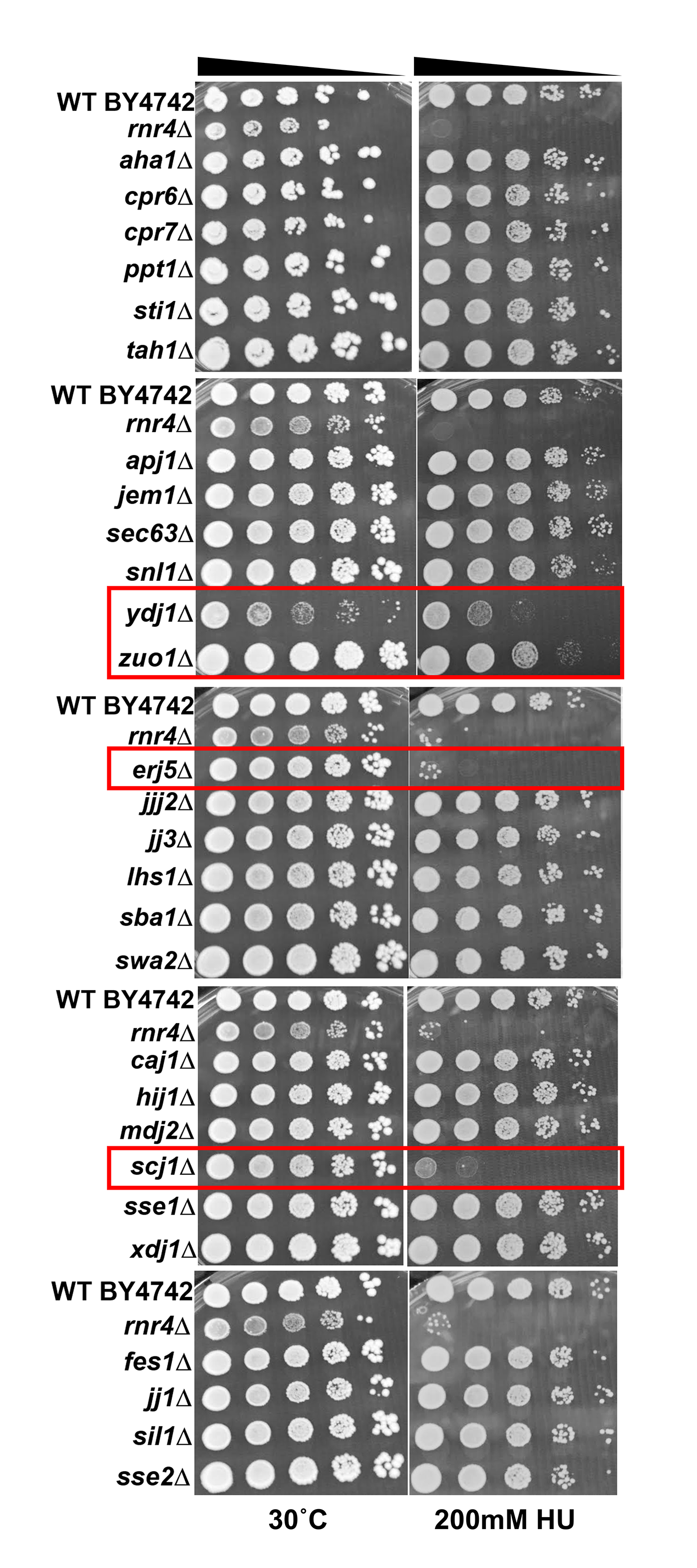

Supplement: S1 Fig — WT BY4742 or BY4742 cells lacking Rnr4 or 28 co-chaperone proteins were grown overnight to saturation and serial 10-fold dilutions were plated by pin plating from 96-well plates onto YPD alone or YPD containing 200 mM HU. Plates were imaged after 3 days. HU-sensitive co-chaperone mutant strains are highlighted in red. (TIF) [file pgen.1007462.s002.tif]

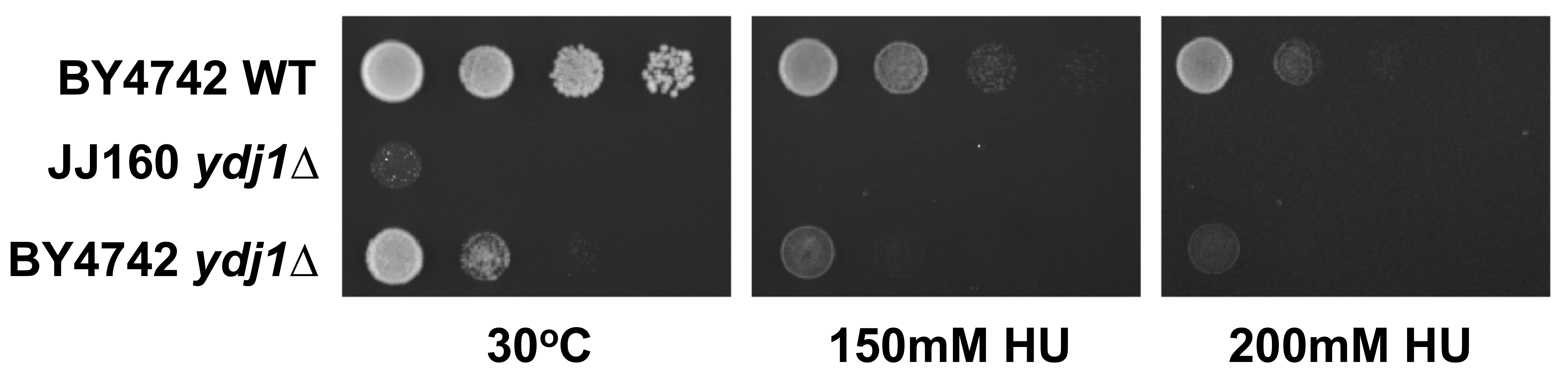

Supplement: S2 Fig — BY4742 WT, BY4742 ydj1Δ and JJ160 cells were grown overnight to saturation and serial 10-fold dilutions were plated by pin plating from 96-well plates onto YPD alone or YPD containing either 150mM or 200 mM HU. Plates were imaged after 3 days. (TIF) [file pgen.1007462.s003.tif]

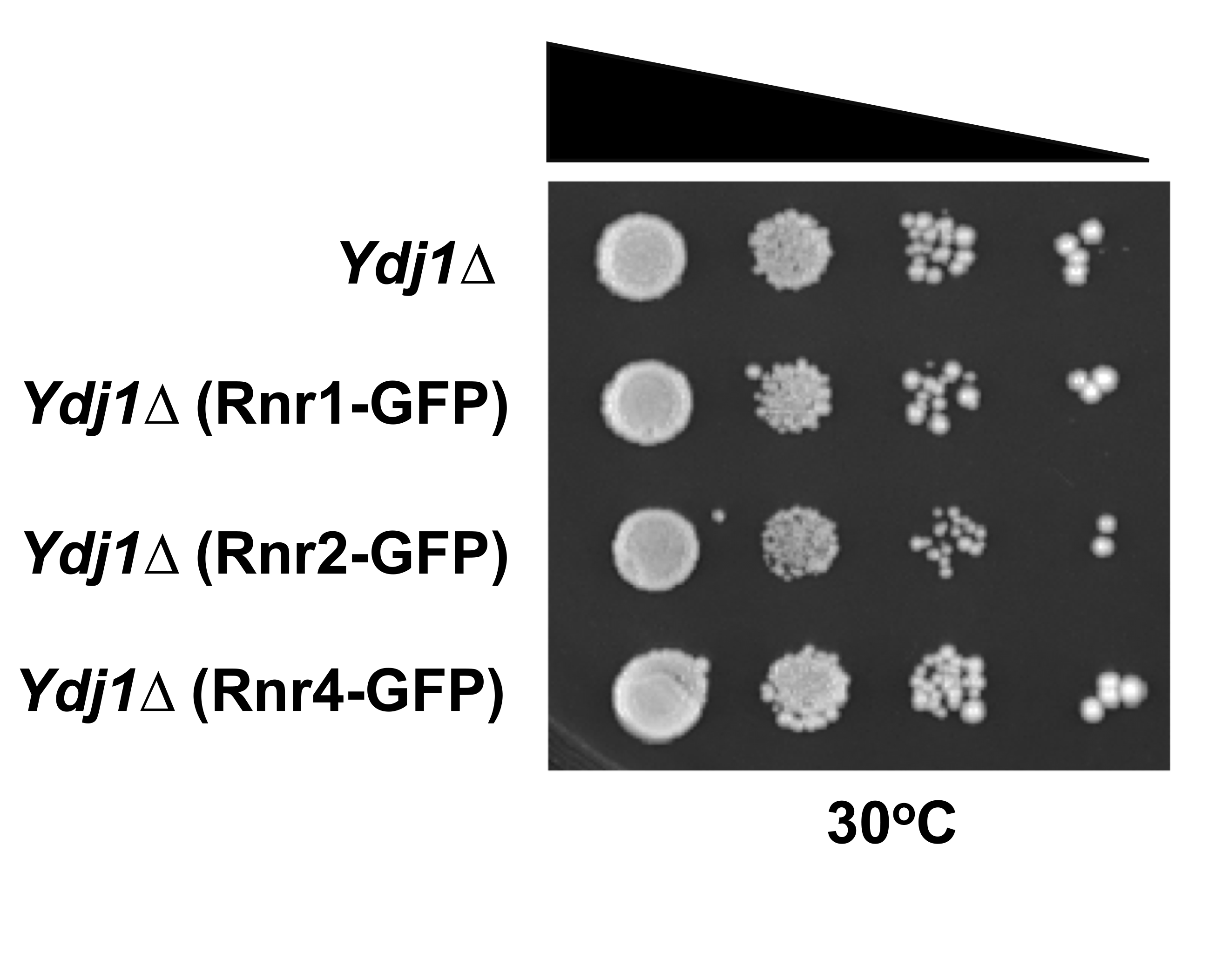

Supplement: S3 Fig — BY4742 WT and BY4742 ydj1Δ cells were grown overnight to saturation and serial 10-fold dilutions were plated by pin plating from 96-well plates onto YPD. Plates were imaged after 3 days. (TIF) [file pgen.1007462.s004.tif]

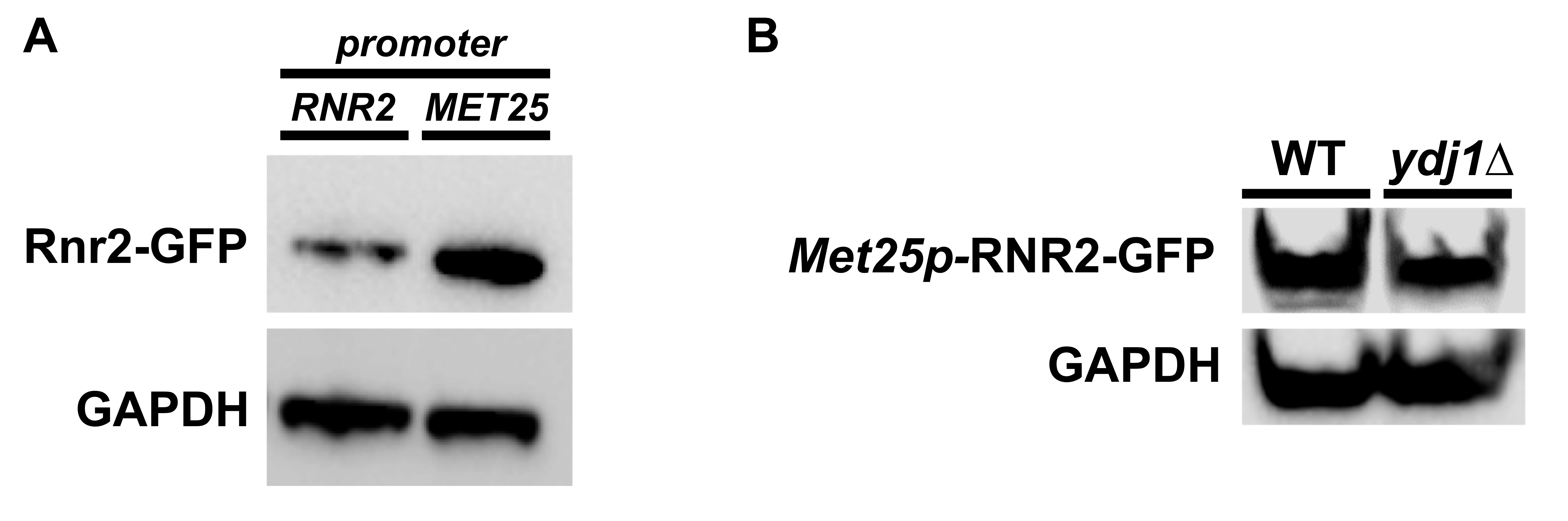

Supplement: S4 Fig — (A) BY4742 WT cells expressing either endogenously tagged Rnr2-GFP or BY4742 WT transformed with plasmid expressing Rnr2-GFP from a constitutively high MET25 promoter. Cell extracts were obtained, resolved on SDS-PAGE gels and analyzed by immunoblotting with anti-GFP and GAPDH antibodies. (B) Even when expressed from a constitutive promoter, Rnr2 levels are lower in ydj1Δ cells. WT and ydj1Δ cells were transformed with a multicopy plasmid expressing Rnr2-GFP from the constitutive MET25 promoter. Extracts were obtained as above, resolved on SDS-PAGE gels and analyzed by immunoblotting with anti-GFP and GAPDH antibodies. (TIF) [file pgen.1007462.s005.tif]
